# Supplementary material for: Non-equilibrium quantum domain reconfiguration dynamics in a two-dimensional electronic crystal and a quantum annealer
Source: Nat Commun. 2024 Jun 6;15:4836. doi: 10.1038/s41467-024-49179-z (PMC11156939; doi:10.1038/s41467-024-49179-z)
Supplement: Supplementary file 1 — Supplementary Information [file 41467_2024_49179_MOESM1_ESM.pdf]

Non-equilibrium quantum domain reconfiguration dynamics in a two-dimensional electronic crystal and a quantum annealer

Supplementary Information

1 Supplementary Notes 1 ... 2
1.1 The experimental setup, domain creation by laser or charge injection through tip ... 2
1.2 Calculation of the STM tip and sample temperature during transformation and scanning ... 2
1.2.1 Thermal behavior of the sample during the creation of the domain state by a single electrical pulse ... 4
1.2.2 Heating of the sample by the tip current during relaxation measurements. ... 4
1.3 Detection of domain reconfiguration ... 5
1.4 Temperature dependence of domain reconfiguration rate ... 8
1.5 Tip current and voltage dependence of domain reconfiguration: measurement details ... 9
1.6 The experiments measuring 'light' and 'dark' relaxation by STM ... 11
2 Supplementary Notes 2 ... 13
2.1 Details of the theoretical model ... 13
2.2 Classical Monte Carlo simulations ... 16
2.3 WKB Approximation and Macroscopic Quantum Tunneling ... 20
2.4 Quantum Kramers theory of metastable decay ... 21
2.5 Details on the incoherent macroscopic tunneling of our model ... 21
3 Supplementary Notes 3 ... 23
3.1 Applying the model on D-Wave's machine ... 23
3.2 Introducing quantum dynamics by reverse annealing ... 25
4 Supplementary Notes 4 ... 27
4.1 Limitations by the annealer on the parameter space ... 27
4.2 Phase diagram calculation details ... 28
4.3 Relaxation measurements ... 30
5 Supplementary References ... 34

1 Supplementary Notes 1

1.1 The experimental setup, domain creation by laser or charge injection through tip

Domains may be created at 4 K either by exposure to a laser pulse[1], or by an electrical pulse from an STM tip[2–4], or by a current pulse passed through a set of electrodes on the surface[4,5]. In this work the domain state was created by using an electrical pulse from the STM tip on a freshly cleaved 1T-TaS_2 sample in UHV. The tip is initially retracted at 50 nm from the sample's surface, then approached with a bias voltage of 5 V and a tunneling current of 10-50 nA. Alternatively, the tip can be approached to the surface with a voltage bias of 0.05 V and a tunneling current of 1-3 nA, then a 4 V electrical pulse of 100 ms duration is applied. The two described procedures generate a domain state area of around 10^4 nm^2. The properties of the resulting domain formation[2,3,6–8], ordering[9] and resistance relaxation as a function of temperature and on different substrates have been reported in detail elsewhere[5].

1.2 Calculation of the STM tip and sample temperature during transformation and scanning

Thermal simulations were performed by solving partial differential equations governing heat conduction using the finite element method (FEM). The thermal model was coupled to the electromagnetic model which calculated the resistive losses (Joule heating) in the contact region: $\rho C_p \frac{\partial T}{\partial t} - \nabla \cdot (k \nabla T) = Q_e$ where $Q_e = \sigma |\nabla V|^2$. The density ρ, heat capacity C_p [10] thermal conductivity k [11], and electrical conductivity σ of all the materials were taken from literature and/or calibrated by experiment[1]. The exact geometry of the W tip, tip mount, tip mount heat sink, 1T-TaS_2 crystal sample and sample holder mount in the STM UHV chamber were reproduced in the model calculations. The 1T-TaS_2 sample ($100\ \mu m$ thick) is glued to the conducting (Mo) sample holder substrate, which is clamped to the cryostat vessel at base temperature (4.5 K) with conducting silver paste. The calculation of temperature of the W tip and 1T-TaS_2 sample for different tunneling currents and tip penetration depths is performed using the COMSOL package. The geometric elements at the tip-sample contact used in the calculation are shown in Supplementary Fig. 1.

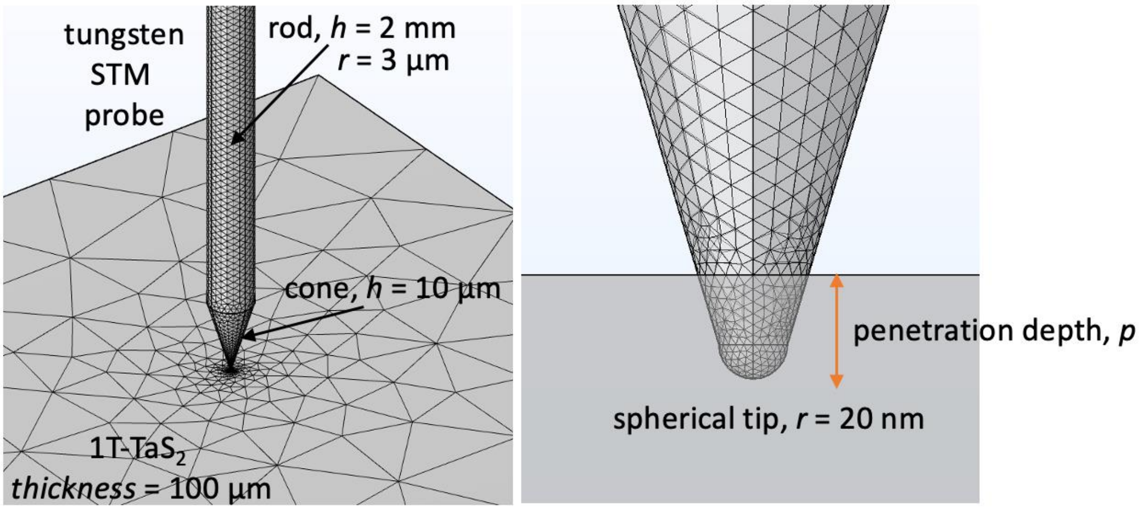

Supplementary Figure 1 | Specification of the geometry in the COMSOL calculations. The tip material is tungsten (W). The base temperature in the calculation is 4.5 K.

The calculations take into account the temperature of the sample and the tip when a current is passing between the tip and the substrate. The model calculation also takes into account:

- the temperature dependence of thermal conductivity, and the specific heat capacity of both 1T-TaS_2 and tip material (W)
- the temperature-dependence and anisotropy of electrical conductivity of both materials. For 1T-TaS_2, the different electrical conductivities of the C and the H phase are taken into account.
- thermal clamping of the tungsten rod at a distance 2 mm to the STM tip mount
- the heat sink of the piezoelectric STM actuator which is in wide-area thermal contact with the tip mount is at base temperature, connected to the cryostat cold finger by multiple copper braids.

The thermal contact between the sample and the sample holder is assumed to be ideal. The calculations are shown in Supplementary Fig. 3 for various penetration depths p. As expected, the behavior beyond a few nm is not very dependent on p.

1.2.1 Thermal behavior of the sample during the creation of the domain state by a single electrical pulse

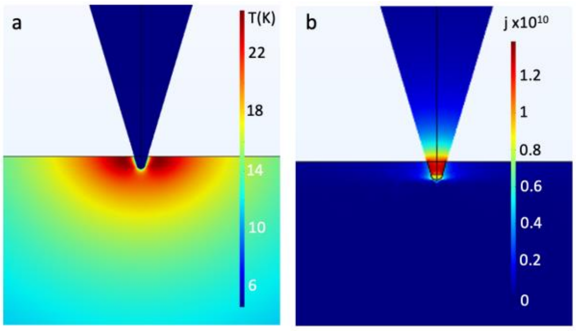

Supplementary Figure 2 | Temperature (a) and current density (b) maps for the strong pulse used to create the domain state. The starting state parameters correspond to the high-resistivity C state. The domain state is examined at ~50 nm from the center. The current density is in units of Amperes/m^3.

A calculation of the tip and sample temperatures in the contact area during single pulse excitation is shown in Supplementary Fig. 2. The maximum temperature at the center reaches ~ 30 K. The temperature drops rapidly away from the contact point. The thermal perturbation is therefore too small to heat the sample through the nearest phase transition to the trigonal (T) or nearly-commensurate (NC) phase. We can safely conclude that the main effect is in response to charge injection, and not thermal.

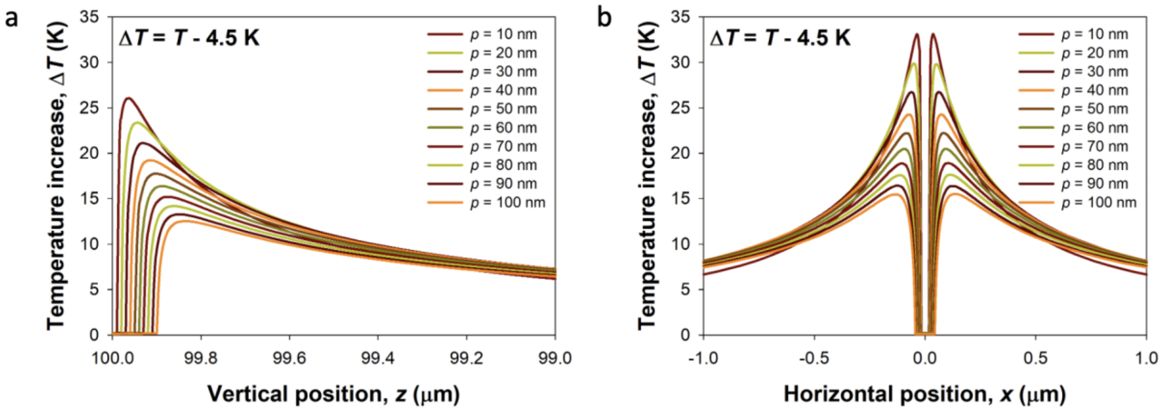

Supplementary Figure 3 | Vertical (a), and lateral (b), temperature profiles within the 1T-TaS_2 sample for different STM tip penetration depths p (see Supplementary Figs. 1,2) resulting from the pulse causing the transformation to the domain state (5V, 50nA). A steady state is assumed, so these are maximum attainable temperatures during the passing of the applied current.

1.2.2 Heating of the sample by the tip current during relaxation measurements.

The temperature calculated for the current and voltage parameters used in the relaxation measurements shown in Fig. 1 of the main text are shown in Supplementary Fig. 4. The maximum calculated temperature rise is $< 0.000001\ K$. We conclude that the temperature rise due to the STM tip currents used in relaxation measurements can be neglected.

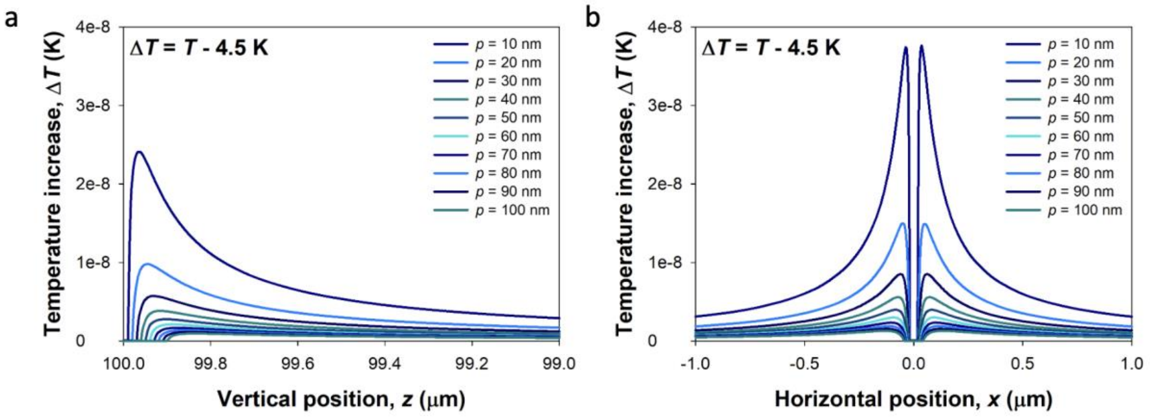

Supplementary Figure 4 | Vertical (a), and lateral (b), temperature profiles within the 1T-TaS_2 sample for different STM tip penetration depths p (see Supplementary Figs. 1, 2) during relaxation measurements using the parameters presented in Fig. 1 of the main text. The maximum temperature rise is < 0.000001 K. The sample and tip mount base temperatures are both 4.5 K.

1.3 Detection of domain reconfiguration

STM measures the local density of states (LDOS) at the point of tunnelling from the STM tip into the sample. In the local state approximation, appropriate for the Wigner crystal state, for state E_i the LDOS is given by[12], $\rho_{local}(E,\mathrm{r}) \propto \sum_{i=1}^{N} |\psi_i(E_i,\mathrm{r})|^2 \delta(E-E_i)$. Integrating $\rho_{local}(E,r)$ over energy in the interval $\pm\Delta/2$, where Δ is the full bandwidth of state E_i, we obtain a real-space electron density $\rho(r)$ for state E_i. The total charge ρ in state E_i is given by the integral over a contour S around a localized state $\int_S \rho(\mathrm{r})d\mathrm{r} = \rho$. If charge is localised, maxima in STM images can be interpreted as an electron count, which can be used to quantify the domain wall reconfiguration. By measuring $\rho(\mathrm{r})$ patterns as a function of time, one obtains a quantitative measure of charge reconfiguration in each frame. We note that this does not imply any single particle motion between frames, but measures the number of sites involved in the domain reconfiguration in a given time interval. Furthermore, the absolute number density is not of interest, only the changes with time. The criterion for domain wall characterization is thus somewhat arbitrary. To obtain an accurate rate it is important that the counting is performed consistently.

In these measurements, the border between the ground state and the domain state is first found. Supplementary Fig. 5 shows an example of such an STM image. An area is typically chosen for analysis with as few imperfections and structural defects (such as the bright yellow spots in Supplementary Fig. 5) as possible. A series of N images of the same area (40x40 nm^2) are taken at regular intervals to record the relaxation of the domain structure (Supplementary Fig. 6). Tip-scanning parameters are -0.8 V for voltage bias, 50 pA for tunneling current and 84 nm/s is the tip speed. The tip scans each line from left to right along the x-axis and then moves to the upper line (y-axis). 8 minutes are required to scan the entire area under these conditions. Images within the same series are rigidly translated referring to a fixed point common to each image (e.g. a stationary polaron) and then uneven borders are cut. This corrects possible shifts of the tip-sample relative position. To compare two consecutive images (i and i+1; with i = 1, . . ., N), we detect the polarons' centers in each image (shown by red dots in Supplementary Fig. 7) by using a blob detection algorithm in Python environment. Then the centers' patterns are juxtaposed (Supplementary Fig. 8a) to count the number of polarons which have moved.

The allowed displacement of the electrons is defined by the internal structure of 1T-TaS_2 polarons, each of which involves thirteen Ta atoms arranged in the shape of a David's star, with 6 Ta atoms on the vertexes, 6 at the corners, and one in the center (Supplementary Fig. 8b). Due to the periodicity of the system, there are only two non-trivial ways to move a polaron, i.e. move the central atom to a corner or to a vertex of the David's star. This corresponds to a minimum polaron's movement of ~0.33 nm. Thus, to determine if a certain polaron moved between two images, we take its coordinates in the two images and we evaluate if they differ by more than 0.33 nm. In the affirmative case, the polaron is considered as moved. Supplementary Fig. 8c shows one possible arrangement of the possible polaron positions at the domain wall enumerated by Ma et al [2]. The movement of a domain is associated with an energy cost ΔE. To first order, $\Delta E \sim N$, where N is the number of polarons involved in the reconfiguration.
The fraction (*f*) of moved polarons is calculated as the number of moved polarons divided by the total number of polarons:

$$f(i, i+1) = \frac{[number\ of\ moved\ polarons\ between\ images\ (i) and\ (i+1)]}{[total\ number\ of\ polarons\ in\ image\ (i)]}. \quad (1)$$

We estimated the error of f as given mainly by polarons which are detected only in one of the two compared images (this usually occurs on the border of the images or inside the domain walls). Its value is calculated as the relative difference between the total detected polarons in the two images:

$$\delta f(i, i+1) = \frac{[|total\ polarons\ in\ (i) - total\ polarons\ in\ (i+1)|]}{[total\ polarons\ in\ (i)]}. \quad (2)$$

The described procedure is repeated for each pairs of consecutive images in a certain series. Finally, summing all the calculated fractions and dividing by the time (*T*) needed to scan the *N* images of the series ($T = N \times 8\ min.$), we obtain the rate *R* of movements in the series:

$$R = \frac{\sum_{i=1\ldots N-1} f(i, i+1)}{T} \quad (3)$$

$$\delta R = \frac{\sum_{i=1\ldots N-1} \delta f(i, i+1)}{T}$$

With this definition, R is always ≤ 1. This is due to the fact that if a polaron moves two (or more) times between two images, it would still be detected as a single event (or 0, if the polaron returns to its starting site). Note that the absolute value of N is of no significance, while the accuracy of the rate measurement is ensured by the use of a consistent algorithm throughout the analysis.

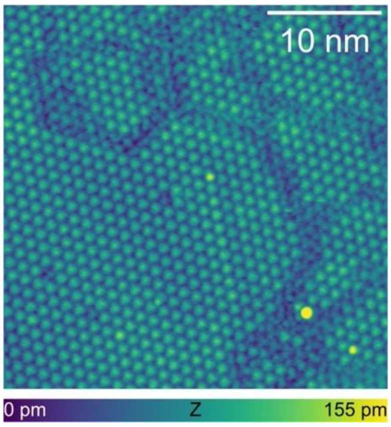

Supplementary Figure 5 | An STM image of the border between the ground state (down/left) and the domain state (up/right) in 1T-TaS_2.

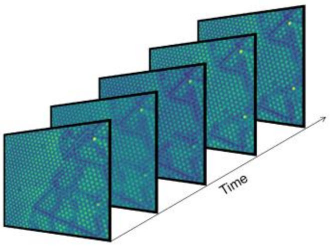

Supplementary Figure 6 | Scheme of a series taken while relaxation of the domain state occurs. Each image shows the same area. Images are separated by 8 minutes.

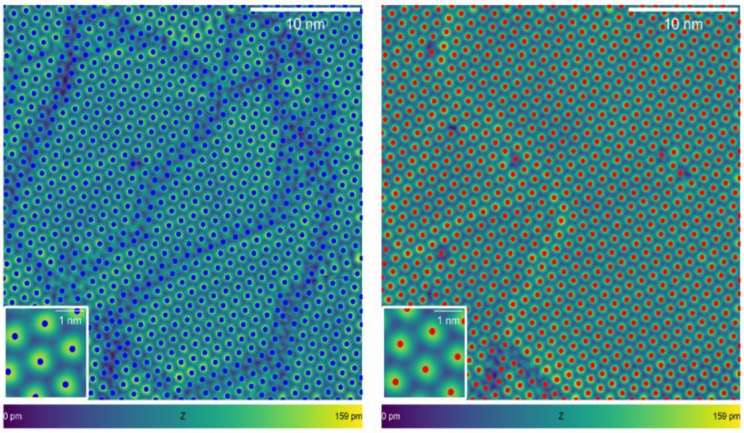

Supplementary Figure 7 | Patterns of polarons in two consecutive images of the same area. The patterns are superimposed to the STM images. In the bottom-left corner of both images the pattern is enlarged.

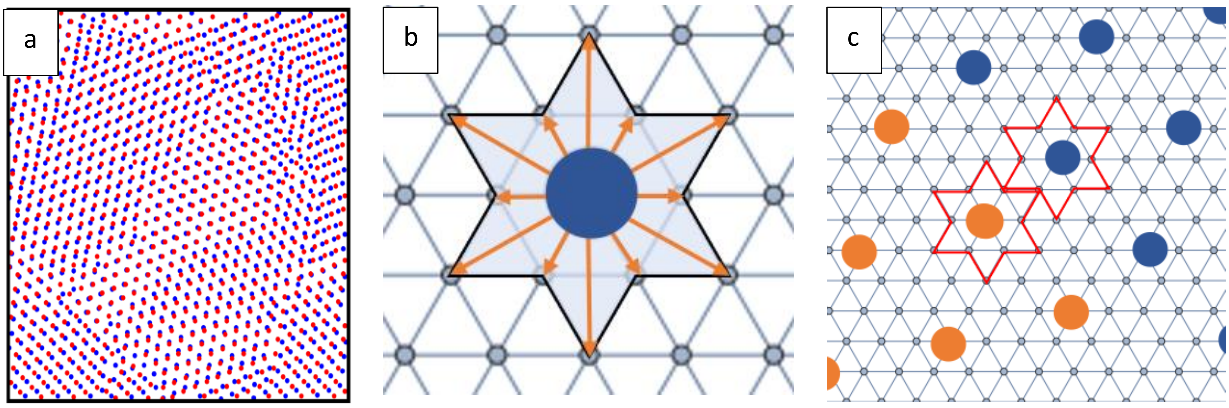

Supplementary Figure 8 | a, Juxtaposition of polarons' centres of images in Supplementary Fig. 7 When the polaron didn't move, the red dot covers the blue dot, otherwise both dots are visible. b, The possible 6 nearest and 6 next-nearest neighbour Ta positions to which an electron can move in a domain reconfiguration. c, An example of a domain wall[2,9], showing explicitly the positions of the polarons at the junction of two domains.

1.4 Temperature dependence of domain reconfiguration rate

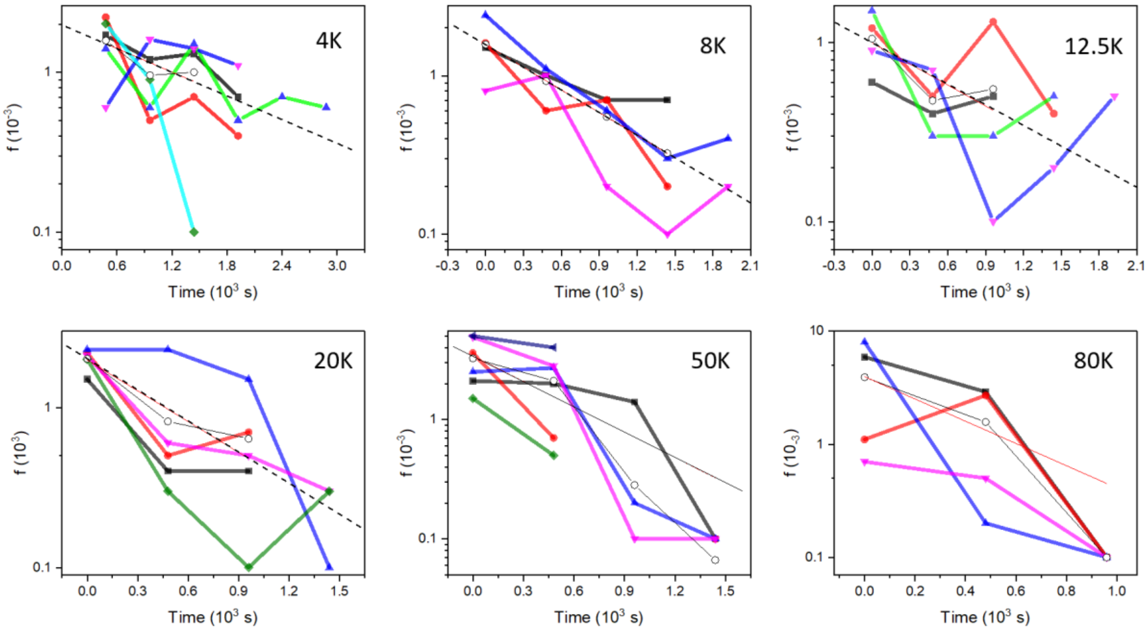

Supplementary Figure 9 | Reconfiguration f of the domain state at 6 different temperatures T.

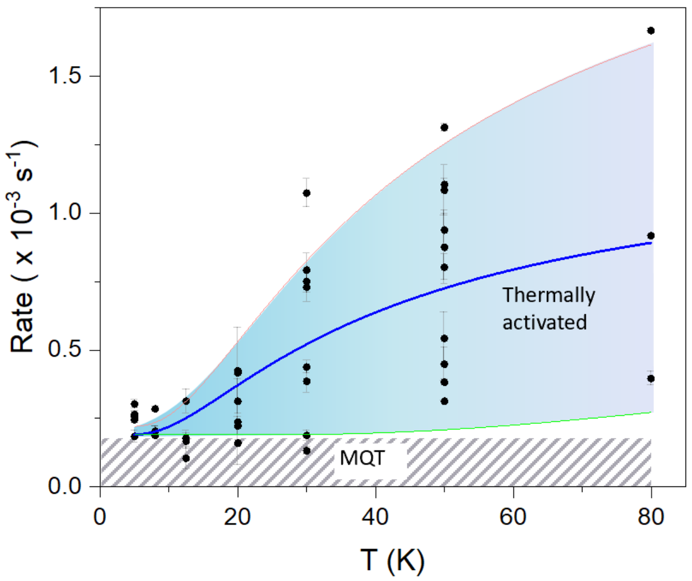

Supplementary Figure 10 | Reconfiguration rate R(T) as a function of temperature with error bars representing counting errors of polaronic reconfiguration events. Experimental runs presented in Supplementary Fig. 9 are shown separately. The fits are described in the text.

Supplementary Fig. 9 shows the raw data on relaxation at different temperatures. The scatter of the data is a result of the fact that domain reconfiguration process is measured on a relatively small area which is comparable to the domain size. Measurement of a larger area would require faster scanning, which in turn would sacrifice resolution of the measurement of polaron motion. The presented measurements are a compromise that optimizes the resolution and measurement area.

Supplementary Fig. 10 shows the data of relaxation as a function of temperature for all measurements individually. Note that the scatter of the data is very small at low temperature, but increases with temperature. As discussed in the main text, the temperature-activated domain relaxation processes have a range of different E_B, reflecting the wide spread of relaxation rates, as defined by the changing energy landscape each time the configuration changes[2,3]. The average rate is presented in Fig. 1 of the main paper. The boundaries of the blue shaded area represent fits to the phenomenological model[13] given in the main text, where $R(T) = R_q + R_0 \exp\left(-\frac{E_B}{k_B T}\right)$ with values of $E_B = 40\,K$ (top curve) and $E_B = 200\,K$ (bottom curve) with $R_0 = 0.0023\,s^{-1}$ and $0.001\,s^{-1}$ respectively. The blue curve is the fit to the average of all the data shown in the main text, giving $E_B = 36\,K$, with $R_0 = 0.0011 s^{-1}$. Note that the low-temperature value for the quantum rate $R_q = 1.9 \times 10^{-4}\,s^{-1}$ is the same in all three fits. The patterned area represents the base quantum rate, which is temperature independent.

1.5 Tip current and voltage dependence of domain reconfiguration: measurement details

The thermal calculation in section 1.2 has shown that during scanning the effect of Joule heating on the sample is negligible. However, the small tunneling currents may transfer sufficient charge to modify the charge configuration state according to the established mechanisms[1,14,15]. To determine this empirically and determine the role of the tip in the relaxation process, we performed systematic measurements with different scanning parameters (I and V) both at 5 K and 50 K.

Exploring first the effect of STM bias voltage and tip-tunnelling current on R, we find that increasing either the tip current, or the tip-to-sample voltage at 5 K results in a slight increase on the relaxation rate (Supplementary Fig. 12a). Supplementary Fig. 11 shows an example of relaxation series at 5 K for 40-fold increased tunneling current 2 nA and -0.8 V, compared with 50 pA and -0.8 V for the sequences presented in Fig. 1 of the main text. The 40-fold increase in tip current (from 50 pA to 2 nA) increases R rather modestly, from ~2.5×10^{-4} s^{-1} to ~4.2×10^{-4} s^{-1} (a factor of ~1.7). Similarly, quadrupling the tip voltage from -0.8 V to -3.2 V and simultaneously increasing the current three-fold from 50 pA to 150 pA results in a similarly modest change of R from ~2.5×10^{-4} s^{-1} to ~5.5×10^{-4} s^{-1} (a factor of 2.2). Supplementary Fig. 12 shows the comparison between the range of all rates measured for normal scanning parameters (50 pA and -0.8 V) and the average rates for two combinations of different parameters (2 nA and -0.8 V; 150 pA and -3.2 V). Thus, the application of huge increases in current or voltage apparently causes very small changes in R at 5 K.

At 50 K, the effect of changing the scanning parameters over the same range becomes undetectable. Supplementary Fig. 12b shows that relaxation rates at 50 K for different parameters (150 pA and -3.2 V; 2.0 nA and -0.8 V) are compatible with the rate at normal scanning parameters (50 pA and -0.8 V) presented in Fig. 1 of the main text. This confirms that the tip-induced relaxation is small in comparison with intrinsic quantum and thermally activated relaxation rates R_q and R_0 respectively.

A possibility exists that external electrical noise influences R. The electrical noise floor within the UHV LT STM is many orders of magnitude below the value required to change the rate, as discussed above, so it is unlikely to cause enhanced relaxation. Cosmic rays are known to cause perturbations in quantum electrical devices, such as detectors and quantum processors (see main text), and they are also likely to trigger domain reconfiguration by locally depositing energy. However, this would be visible in the STM scans in the form of spikes. These are not very common on our apparatus, and cannot be correlated with relaxation, so at present cannot be identified as an external stimulus that drives relaxation.
The presented data lend support to the arguments presented in the main text (and in the next section, 1.6) based on tunnelling arising from retracted-tip measurements (Fig. 1f). We emphasize that for the purpose of this study it is not important to eliminate completely external relaxation triggers, but only to ascertain that a finite intrinsic rate of domain reconfiguration is present. From our experiments presented above we can safely conclude that while the tip field or current and other external processes may enhance relaxation, a significant part of R_q arises from quantum processes that are intrinsic, and independent of external stimuli.

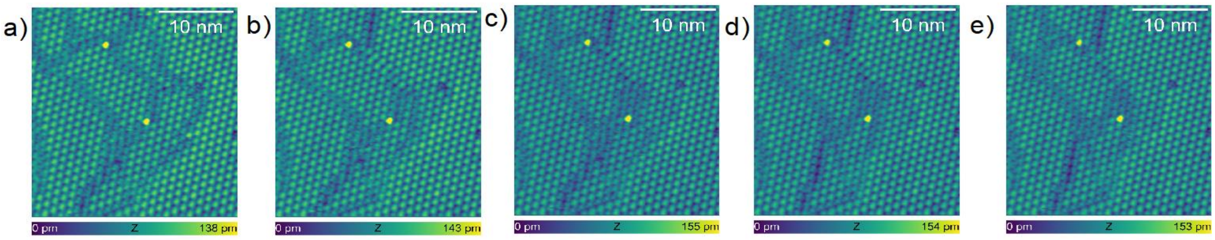

Supplementary Figure 11 | Relaxation of the domain state at 5 K with scanning parameters 2 nA and -0.8 V.

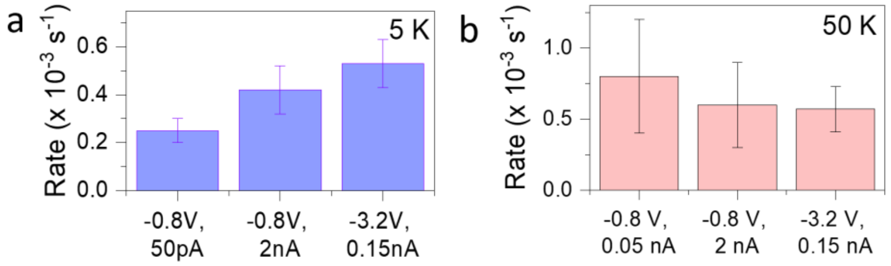

Supplementary Figure 12 | a, Relaxation rates for different parameters at 5 K. The first bar contains all measured rates for normal scanning parameters (50 *pA* and -0.8 *V*). Second and third bars are the average rates and relative errors for different parameters (2 *nA* and -0.8 *V*; 150 *pA* and -3.2 *V*). b, Relaxation rates for different parameters at 50 K. The first bar contains all measured rates for normal scanning parameters (50 *pA* and -0.8 *V* Second and third bars are the average rates and relative errors for different parameters (2 *nA* and -0.8 *V*; 150 *pA* and -3.2 *V*).

1.6 The experiments measuring 'light' and 'dark' relaxation by STM

A series with a long pause between images (dark series) were measured at 5 K to further investigate the role of the tip in the relaxation process. The procedure consists of scanning a first image (i=1) (which requires 8 minutes), then retract the tip several nm away from the surface, wait 32 minutes ("dark relaxation") and finally start scanning a normal "light" series with 8 minutes of scanning time between images ($i = 2, \ldots, N$). Supplementary Fig. 13 shows the scheme of the dark relaxation series.

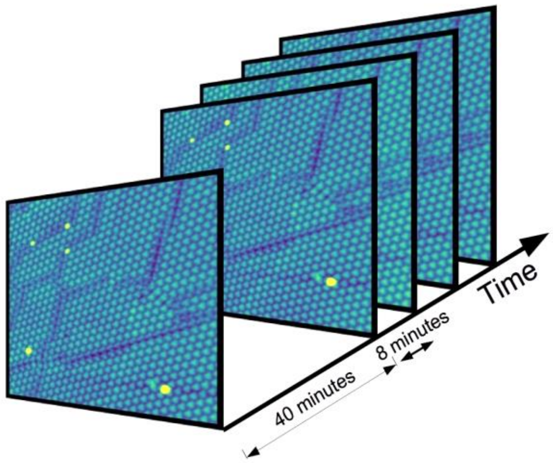

Supplementary Figure 13 | Scheme of a "dark" relaxation series.

The fraction *f* of the moved polarons between consecutive images is then calculated as discussed in section 1.3 for all pairs of consecutive images.

Supplementary Fig. 14a shows an example of a dark series with its relative centers' patterns, while the results for three different dark series are summarized in Supplementary Fig. 14b. As can be seen from the pattern and from the plot, the fraction of moved polarons is consistently larger than the fraction between all other pairs of images. This suggests that the system relaxes to a new configuration during the first 8+32 minutes (with no tip scanning) and then doesn't change significantly thereafter.

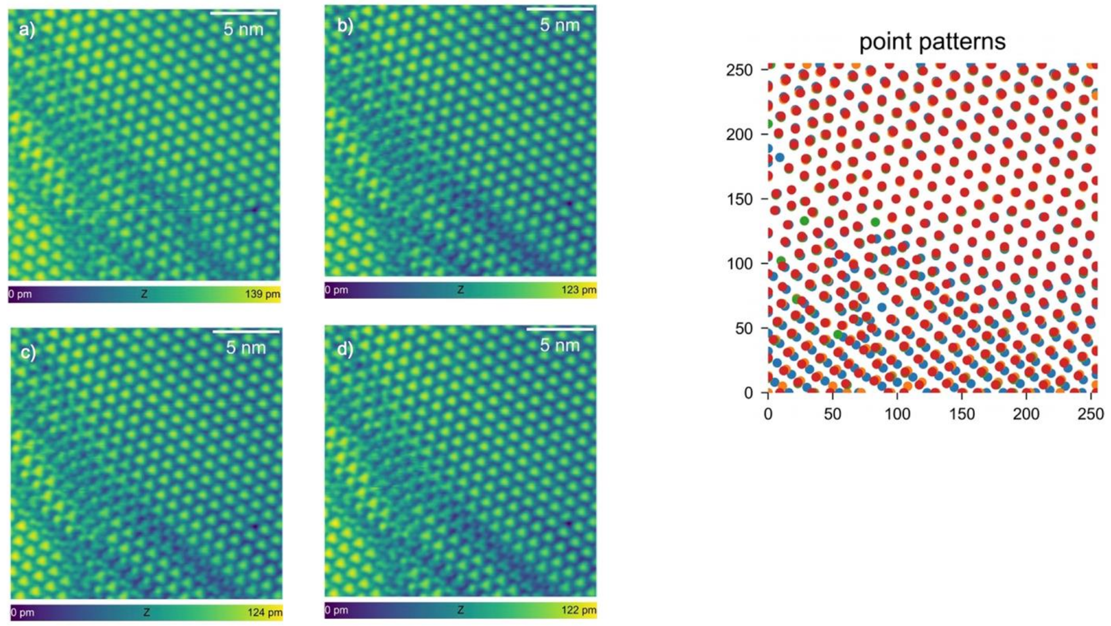

Supplementary Figure 14 | Dark series and relative centers' pattern. Left panel: a, initial image at time = 0 (blue in the pattern), b, after 8 minutes of scanning + 32 minutes of not scanning (orange in the pattern), c, subsequent 8 minutes of scanning (green in the pattern) d, subsequent 8 minutes of scanning (red in the pattern). The right panel shows all four patterns superimposed.

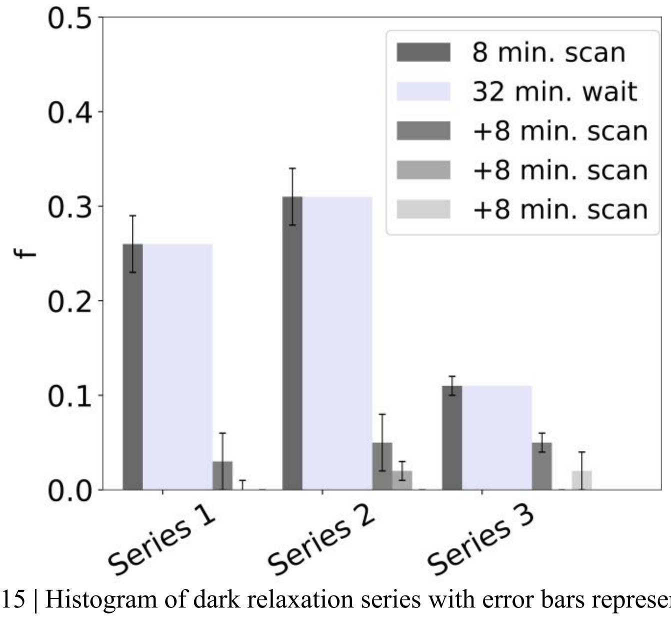

Supplementary Figure 15 | Histogram of dark relaxation series with error bars representing counting errors in measuring polaronic reconfiguration events.

The average fraction of moved polaron during the dark relaxation (8 minutes of scanning *S* +32 minutes of not scanning *NS*) was then compared with the average fraction of moved polarons between images with light relaxation (8 minutes of scanning S) taken in this work (and

discussed in section 1.3) at the same scanning conditions (5 K, -0.8 V for tip-sample voltage bias, 50 pA for tunneling current). A histogram showing the polaron fraction moved in a number of repeated measurements is shown in Supplementary Fig. 15.

In order to estimate the tip-induced effects on the obtained results, the average fraction of moved polarons during dark relaxation was compared to the one measured during regular observation. The comparison shows that:

$$\frac{f_{ave}\,(8\,S + 4{*}8\,S) - f_{ave}\,(8\,S + 32\,NS)}{f_{ave}\,(8\,S + 32\,NS)} \ll 1. \tag{4}$$

This suggests that the fraction of polaron moved during the 8 + 32 minutes of dark relaxation can mostly be attributed to non-tip-induced processes. We conclude that domain reconfiguration takes place at a similar rate in the absence of tip scanning as with tip scanning. The tip thus has a relatively minor effect.

A fit to the 'dark' reconfiguration rate is shown in Fig. 1f of the main text, on the same time-line as for 'light' measurements where a scan takes place continuously. The rates are comparable.

2 Supplementary Notes 2

The low-temperature charge ordering in 1T-TaS_2 is considered to be in the lattice Wigner crystal (WC) limit, which occurs when the electronic Coulomb energy significantly exceeds their kinetic energy[16]. The criterion for Wigner crystallization is defined in terms of the dimensionless Wigner-Seitz radius $r_s = V/T$, where V is the Coulomb energy and T is the kinetic energy. For a 2D electron system, $r_s = e^2 m/(\hbar^2 n^{1/2})$, where n is the electron density, e is elementary charge, and m is the effective mass of electron. In 1T-TaS_2, r_s is enhanced by the small carrier concentration (one electron per 13 unit cells - see Fig. 1a) and due to strong coupling to the lattice, polaronic effects renormalize the electronic bands, resulting in an increase of the effective mass. Altogether, this leads to a large expected r_s, and WC physics[16]. Doping, external charge injection[4,5,7,8] or optical perturbation[1,5] introduces additional charges in the WC, which leads to a complex phase diagram with the formation of domains, discommensurations[5] and charge fractionalization[14,16] (Fig. 1b). The resulting domain wall structure allows multiple possible configurations, arising from different relative domain positions, each of which is a well-defined macroscopic quantum state in the occupation number basis, separated from each other by a potential energy barrier. Such DWs arising from electron "overcrowding" have been observed experimentally by STM in numerous transition metal dichalcogenides[16]. 1T-TaS_2 is of particular interest because the domain reconfiguration dynamics can be tuned by temperature[5] such that at sufficiently low temperatures, the domain reconfigurations are slow enough to be recorded by scanning tunneling microscopy (STM).

2.1 Details of the theoretical model

In this chapter we introduce our theoretical model of 1T-TaS_2 already developed and explored in our previous work[16]. We present its classical charge ordering aspects and their relevance to the simulation on D-Wave's quantum computer. The Hamiltonian presented in this section is mapped onto the D-Wave Hamiltonian H_{DW}.

At the Fermi level, 1T-TaS_2 exhibits a sparsely populated (1 e^- per 13 unit cells) narrow single Ta band. According to[17], chapter 4.2, we choose an appropriate model of an interacting single electron band within the tight-binding approximation

$$H = \sum_{i,j,s,s'} (t_{i,j} - \mu\delta_{i,j})\delta_{s,s'} c_{i,s}^{\dagger} c_{j,s'} + \sum_{\mathrm{q},i,s} \omega_{\mathrm{q}} n_{i,s} (u_{i,s}(\mathrm{q}) d_{\mathrm{q}} + h.c.) + \frac{1}{2}\sum_{i,j,s,s'} V_c(i,j) n_{i,s} n_{j,s'} + \sum_{\mathrm{q}} \omega_{\mathrm{q}} (d_{\mathrm{q}}^{\dagger} d_{\mathrm{q}} + 1/2), \quad (5)$$

where $t_{i,j}$ is the hopping integral between lattice sites i and j, μ the chemical potential, $c_{i,s}$ the annihilation operator of an electron at site i with spin s, $n_{i,s} = c_{i,s}^{\dagger} c_{i,s}$. Electrons interact with each other via Coulomb interaction $V_c(i,j)$, as well as with phononic degrees of freedom, where d_{q} is the annihilation operator of a phonon with wave vector q and ω_{q} is it's frequency. The electron-phonon interaction is taken to be Fröhlich like and it's matrix element is $u_{i,s}(\mathrm{q})$ and $\hbar = 1$. In the strong electron-phonon interaction limit, H can be solved exactly via the Lang Firsov canonical transformation ($\widetilde{H} = e^S H e^{-S}$, $S = \sum_{\mathrm{q},i,s} n_{i,s} (u_{i,s}(\mathrm{q}) d_{\mathrm{q}} - h.c.)$), where electrons get dressed by a surrounding lattice deformation and therefore become quasi particles dubbed as polarons. After the transformation H becomes

$$\widetilde{H} = \sum_{i,j,s,s'} (T_{i,s,j,s'} - \mu\delta_{i,j})\delta_{s,s'} \tilde{c}_{i,s}^{\dagger} \tilde{c}_{j,s'} - E_p \sum_{i,s} \tilde{n}_{i,s} + \frac{1}{2}\sum_{i,j,s,s'} v(i,s,j,s') \tilde{n}_{i,s} \tilde{n}_{j,s'} + \sum_{\mathrm{q}} \omega_{\mathrm{q}} (\tilde{d}_{\mathrm{q}}^{\dagger} \tilde{d}_{\mathrm{q}} + 1/2), \quad (6)$$

where $\tilde{c}_{i,s} = c_{i,s} \exp(\sum_{\mathrm{q}} u_{i,s}(\mathrm{q}) d_{\mathrm{q}} - h.c.)$ is the annihilation operator of a polaron and $\tilde{d}_{\mathrm{q}} = d_{\mathrm{q}} - \sum_{i,s} n_{i,s} u_{i,s}^{*}(\mathrm{q})$ is the new displaced phonon annihilation operator. The polaronic hopping integral is now $T_{i,s,j,s'} = t_{i,j} \exp(\sum_{\mathrm{q}} [u_{i,s}(\mathrm{q}) - u_{j,s'}(\mathrm{q})] d_{\mathrm{q}} - h.c.)$, the polaron-polaron interaction is $v(i,s,j,s') = V_c(i,j) - 2\sum_{\mathrm{q}} \omega_{\mathrm{q}} (u_{i,s}(\mathrm{q}) u_{j,s'}^{*}(\mathrm{q}))$ and E_p is the polaronic binding energy. In the strong electron-phonon interaction limit, the hopping integral of polarons can be neglected, which makes $\widetilde{H}$ diagonal in the polaronic occupation number basis and is therefore exactly solved. Here, we focus on the configurational ordering of polarons due to the interaction between them. Therefore, we consider them as spinless charged particles on a triangular lattice.

We rewrite $\widetilde{H}$ with the classical 2D interaction Hamiltonian

$$H_{int} = \frac{1}{2}\sum_{i,j}^{N} V(i,j) q_i q_j - \mu \sum_{i}^{N} q_i, \quad (7)$$

where $V(i,j) = V_0 \exp(-r_{i,j}/l_s)/r_{i,j}$, l_s is the screening radius, $r_{i,j} = |\mathrm{r}_i - \mathrm{r}_j|$, r_i is the i-th out of N lattice sites, $q_i \in \{0,1\}$ is the occupation number of lattice site i and the sum runs over all lattice sites. The number of polarons in the system is varied by the chemical potential μ. Polarons are considered as screened point charges that can only reside on Ta sites of the triangular atomic lattice. In this work we considered a triangular lattice with 2008 sites and open boundary conditions, where polarons repel each other via nearest neighbor repulsion. μ was set to a fixed value for which the ground state of H_{int} is a 1/3 polaronic lattice[16].

H_{int} describes the domain structure and salient features of the phase diagram very well[16,18], but not quantum dynamics. To introduce quantum tunneling between different configurations of domains we first simplify H_{int} to $H'_{int} = \sum_{i,j}^{N} Q_{i,j} q_i q_j$, where $Q_{i,j} = \frac{1}{2} V(i,j) - \mu\delta_{i,j}$. This makes $\widetilde{H}$ applicable to the quadratic unconstrained binary optimization formalism implemented

on the D-Wave "Advantage" quantum processor, with > 5000 physical qubits. The Hamiltonian that is simulated on the device is the transverse field Ising model (TFIM)

$$H_{DW} = -\frac{A(t)}{2}\sum_i \sigma_i^x + \frac{B(t)}{2}\left(\sum_{i<j} J_{i,j}\sigma_i^z\sigma_j^z + \sum_i h_i\sigma_i^z\right), \tag{8}$$

where $\sigma_i^{x,z}$ are Pauli matrices operating on qubit q_i, $J_{i,j}$ are couplings between qubits q_i and q_j and h_i is the longitudinal external field exerted upon q_i. In order to simulate 1T-TaS$_2$ with D-Wave's quantum computer, we map H'_{int} onto H_{DW} by applying the transformation from spin to qubit variables $\sigma_i^z = 2q_i - 1$. Consequently, $h_i = \sum_{j=i}^{N} Q_{i,j}/2$ and $J_{i,j} = Q_{i,j}/4$, which reduces H'_{int} to the second term of H_{DW}. The σ^x term in H_{DW} describes tunneling between different configurational states and with this additional term our simulated model can also be viewed as an extended two-dimensional TFIM on a triangular lattice. The triangular lattice with 2008 sites combined with the correlated structure of H_{int} requires a specific embedding which uses 2673 qubits subject to the TFIM. Details on the embedding can be found in a subsequent chapter.

In order to make the connection of our model as deployed on D-Wave and a Wigner crystal, we interpret the σ^x term as the kinetic energy of polarons. Using the 2D Jordan Wigner transformation

$$\sigma_i^{\pm} = \sigma_i^x \pm i\sigma_i^y;\ \sigma_i^- = a_i \exp(i\alpha_i);\ \sigma_i^+ = a_i^\dagger \exp(-i\alpha_i); \tag{9}$$

$$\alpha_i = \pi\left(\sum_{x=1}^{i_x-1}\sum_{y=1}^{N_y} q_{x,y} + \sum_{y=1}^{i_y-1} q_{i_x,y}\right); q_i = q_{i_x,i_y} = a_i^\dagger a_i,$$

where σ_i^+ is the spin raising, σ_i^- spin lowering operator and a_i the annihilation operator of the spinless polaron or qubit, H_{DW} becomes

$$H'_{DW} = -\frac{A(t)}{4}\sum_i\left(a_i^\dagger e^{-i\alpha_i} + a_i e^{i\alpha_i}\right) + \frac{B(t)}{2}\sum_{i<j} Q_{i,j}q_iq_j\,. \tag{10}$$

In the limit of strong interactions, domain walls dominate the system and the kinetic energy term simplifies into a hopping term for the domain walls $\sum_{\langle ij\rangle} t_{ij}a_i^\dagger a_j + h.c.$[19]

The Wigner-Seitz radius r_s in our case is therefore related to the ratio between the second and the first term. The kinetic energy has an anomalous form and can be interpreted as the creation or annihilation of a spinless polaron at site i, which depends on the current polaronic configuration. The comparison between the effect of this form of kinetic energy and the conventional hopping form is beyond the scope of this work and remains an interesting question for future studies.

In this paper we use an analogue to the $1/13$ electronic lattice which forms in TAS. We use a $1/3$ electronic lattice due to the smaller system sizes considered here. We have shown already in[16] that classically the system behaves very similar to the $1/13$ case albeit at a higher polaron density. We assume here that this generalizes also to the quantum version of this model, which is considered in this work.

In order to capture non-equilibrium quantum domain wall reconfiguration dynamics, we require at least a few hundred atomic sites or qubits, which is impossible with state-of-the-art classical simulation methods and hardware[20]. Another promising candidate are quantum Monte Carlo simulations with added noise, which are not developed for directly probing non-equilibrium

dynamics and it is still an open question whether they could be applied in our case. We also checked whether classical Monte Carlo simulations (next section) in the presence of a thermal bath can predict a crossover in $R(T)$. As expected, thermally activated processes are responsible for the T-dependent reconfiguration rate. However, they cannot explain the T-independent behavior we observe in 1T-TaS_2 at low T for interaction potentials known from previous literature[14,21,22].

2.2 Classical Monte Carlo simulations

We performed classical Monte Carlo (MC) simulations in the form of simulated annealing, analogous to the quantum annealing performed on the D-Wave Advantage. Our classical simulations consist of Markov chain dynamics with the Hamiltonian $H_{int} = \frac{1}{2}\sum_{i,j}^{N} V(i,j)q_i q_j - \mu \sum_{i}^{N} q_i$, on a triangular lattice using the standard Metropolis algorithm. The proposed successive state in the chain consists of switching the positions of a particle and a hole on two randomly selected nearest neighboring sites. After this step, we also select a random site and destroy a particle if it's occupied or create one if it's empty. The simulations were performed at a fixed value of $\mu = V_0$ on a triangular lattice with 2008 sites and lattice spacing a, depicted in the section "Applying the model on the D-Wave's machine". The interaction potential $V(i,j)$ is a positive constant V_0 when i and j are nearest neighbors and, in a subsequent case, also next-nearest neighbors. Simulated annealing was performed by decreasing the effective temperature $T_{eff} = k_B T/V_0$ from 0.5 to 0.001 in a 100 steps with 1000 MC sweeps at each temperature point. Our system exhibits an ordering phase transition from a high-temperature disordered gaseous phase to a crystalline ordered phase at low temperature. Supplementary Fig. 16 shows snapshots of the simulated annealing process at 4 different values of T_{eff}. The left-most image is far above the phase transition, where the polaronic configuration is essentially random. The next two images toward the right are close above and below the phase transition temperature. The right-most image shows the final state, which is a striped ordered state. This is not the ground state of the model, which is a uniform blue lattice. The reason why we don't observe the ground state is due to the fact that the domain walls cost zero energy in terms of interaction. However, the energy could be lowered still with the addition of more polarons in the domain walls and thereby decreasing the chemical potential term. When the simulation crosses the phase transition, bubbles of the 3 possible ground state lattices begin to form and inevitably produce domain walls. After their formation they are very hard to get rid of from the point of view of simulated annealing, due to their low energy cost. For example, the final state in Supplementary Fig. 16 has an energy per particle (if $V_0 = 1$) -0.7316 compared to the -0.9895 in the case of the same Hamiltonian being applied to the right-most configuration in Supplementary Fig. 18.

Despite our faliure of finding the ground state using MC simulations, it is still important to investigate this specific instance of the Hamiltonian and observe if the rate of polaronic movements $R(T_{eff})$ can explain the experimentally observed rate. Supplementary Fig. 17 shows the average $R(T_{eff})$ during the MC simulation. There is an apparent phase transition at $T_c \approx 0.19$ when the slope of the rate changes abruptly. Also, the rate saturates above the phase transition due to the system effectively sampling from random polaronic configurations. It

saturates at low T_{eff} due to the system's finding a local minimum from where it cannot escape. There are are a few similarities and key differences between the MC simulations, D-Wave simulations and the 1T-TaS_2 experiment:

(i) There is a saturation above the phase transition in both the MC and D-Wave simulations due to melting of the ordered phase and thereby sampling from effectively random configurations. There is no such saturation present in 1T-TaS_2 because the crystalline order is never melted there.

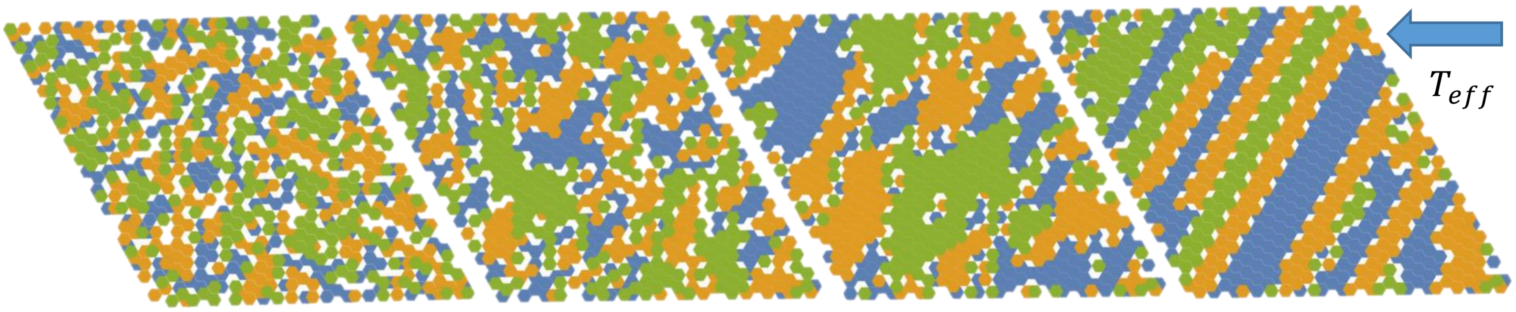

Supplementary Figure 16 | Snapshots of polaronic configurations with nearest neighbor repulsion at different T_{eff}, which increases from right to left as shown on the image.

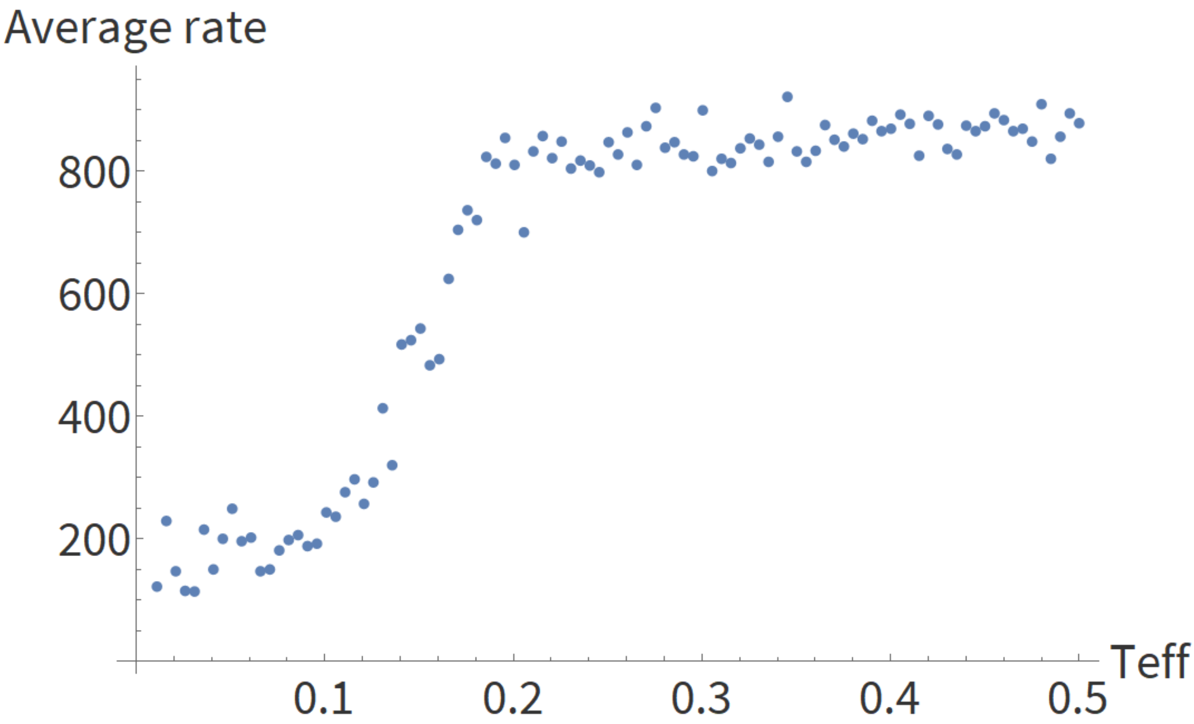

Supplementary Figure 17 | Average rate of polaronic movements measured during the nearest neighbor repulsion Monte Carlo simulation versus T_{eff}. The phase transition at $T_c \approx 0.19$ is obvious from the sharp change of slope in the rate. There is a saturation of the rate at both high and low T_{eff}.

(ii) There is a saturation at low temperatures in all 3 cases, but for very different reasons. In MC simulations it occurs due to the exact zero energy cost of moving a domain wall. This is an artefact of a nearest neighbor repulsion simulation. It is very unlikely that the repulsion in 1T-TaS_2 is only nearest neighbor. In fact, previous work has shown that this is not the case[14,16]. As soon as we turn on next-nearest repulsion with magnitude V_0, we observe the saturation of the rate to the value 0 as we would expect from classical simulations. Supplementary Figs. 18 and

19 show the polaronic snapshots of such a simulation and its $R(T_{eff})$, respectively. The saturation in 1T-TaS2 and D-Wave simulations are of a different origin. They come from incoherent macroscopic quantum tunneling processes, which is discussed in the main text.

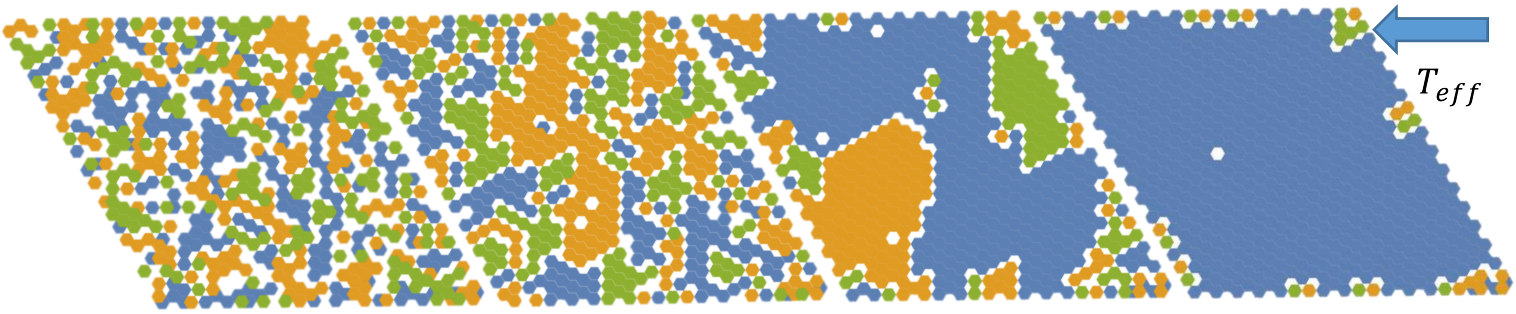

Supplementary Figure 18 | Snapshots of polaronic configurations with next-nearest neighbor repulsion at different T_{eff}, which increases from right to left as shown on the image.

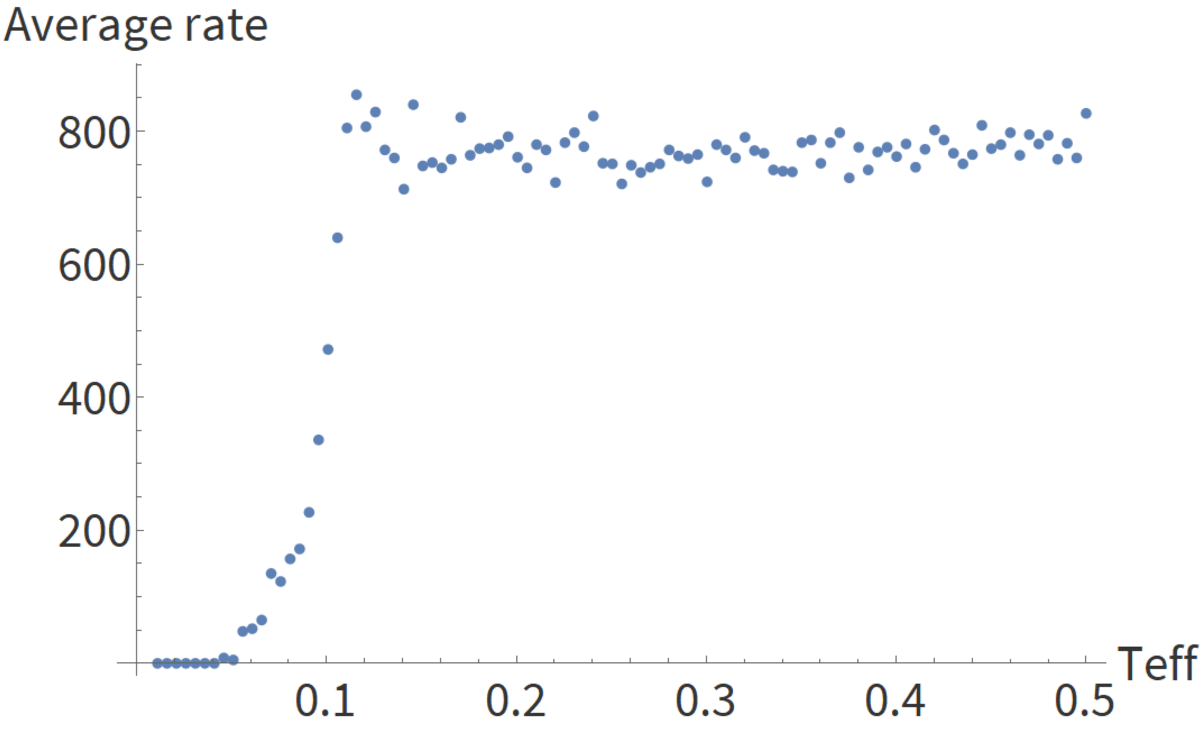

Supplementary Figure 19 | Average rate of polaronic movements measured during the next-nearest neighbor repulsion Monte Carlo simulation versus T_{eff}. The phase transition at $T_c \approx 0.12$ is obvious from the sharp change of slope in the rate. There is a saturation of the rate at both high and low T_{eff}.

(iii) The final difference is in the slope of $R(T)$ below the phase transition. Supplementary Figs. 20 and 21 show the Arrhenius law fit to the average rate in both cases of MC simulations.

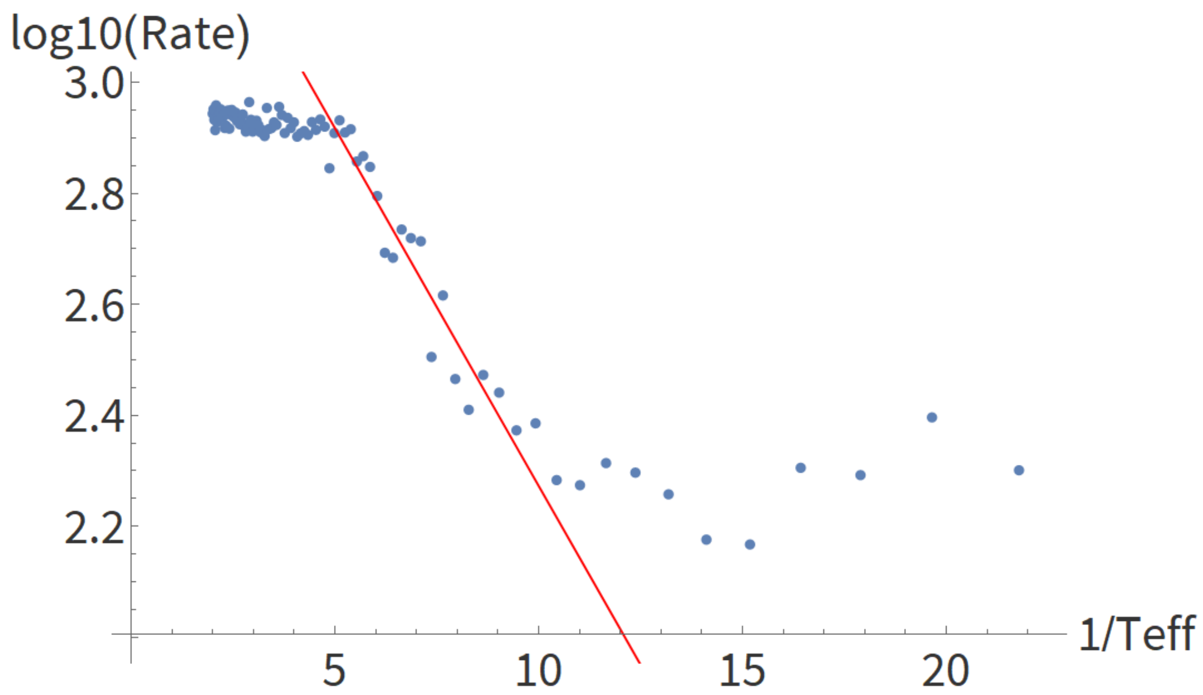

Supplementary Figure 20 | Logarithm of the average rate in the nearest neighbor repulsion Monte Carlo simulation versus $1/T_{eff}$. The slope of the red Arrhenius fit shows a dimensionless energy barrier $\frac{E_B^{NN}}{V_0} = 0.3 \pm 0.2$.

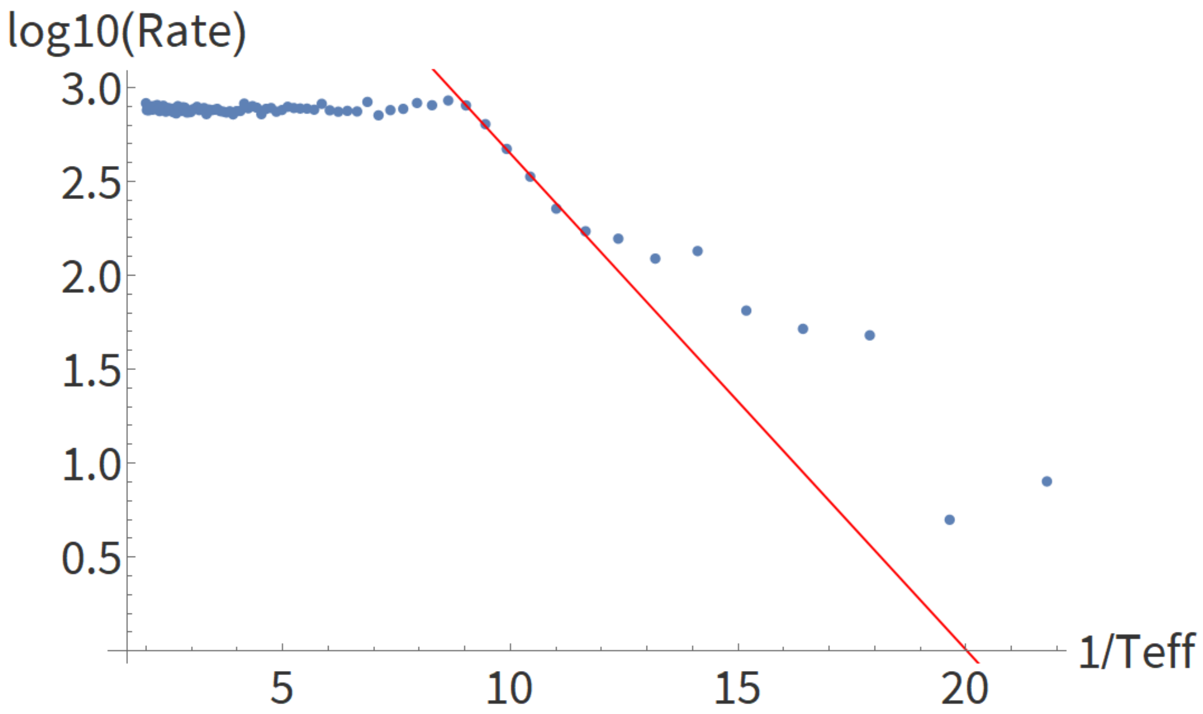

Supplementary Figure 21 | Logarithm of the average rate in the next-nearest neighbor repulsion Monte Carlo simulation versus $1/T_{eff}$. The slope of the red Arrhenius fit shows a dimensionless energy barrier $E_B^{NNN}/V_0 = 0.6 \pm 0.2$.

The average dimensionless energy barrier is twice as high in the next-nearest neighbor repulsion than in the nearest neighbor case, which is consistent with a stronger repulsion potential. We cannot compare this gap to the experiment because we do not know the energy scale for the

polaronic dynamics a priori. However, we can compare it with D-Wave simulations. We obtain them from plots like the one shown in Fig. 2e shown in main text and Supplementary Fig. 33. The dimensionless gaps are $\delta_{r=1} = 3.5 \pm 0.3$; $\delta_{r=2} = 5.3 \pm 0.9$; $\delta_{r=5} = 7.6 \pm 0.9$; $\delta_{r=10} = 13.8 \pm 0.5$; $\delta_{r=15} = 17.2 \pm 1$, where the subscript denotes the value of r in the simulation. The value increases with r as we would expect when the kinetic energy of polarons is reduced compared to the potential energy. The corresponding MC slope can be found in the region $r < 1$, which is inconsistent from the extrapolated value of $r = 58$ in the main text. Therefore, we cannot compare the results obtained from D-Wave simulations with results from MC simulations, which makes sense as the former is a quantum version of the latter.

In conclusion, we have found that MC simulations are highly inconsistent with experimental observations. The main reason is in the simple fact that a classical rate generally saturates to the value 0 under a certain temperature, and the quantum rate doesn't. We have shown that there is in principle a way for the classical rate to be non-zero for any temperature, but this is only possible in the specific case when the energy cost for movement is exactly 0. This is inconsistent with previous work done on 1T-TaS_2. We have also shown that the quantum model deployed on D-Wave cannot be reproduced with MC simulations because the phase transition characteristics do not match.

2.3 WKB Approximation and Macroscopic Quantum Tunneling

For coherent quantum tunneling, which we do not expect to observe in either system, the WKB rate, for $E_b > k_B T$ is given by:

$$R_q = \nu \exp\left(-2w\sqrt{2m^* E_b/\hbar^2}\right)$$

where w is the width of the barrier, and m^* is the effective mass of the object. The physical meaning of the attempt frequency ν is related to the tunneling of the entire object (domain) of mass m^* from one configuration to another in which all electrons change position by $\sim 0.3\ nm$. ν it may be estimated by the energy at which the oscillatory dynamics of a domain patch crosses over from thermally activated to quantum, and the thermal energy becomes comparable to the energy of a quantum oscillator $k_B T \sim \frac{1}{2} m^* \omega^2 x^2$. The reconfiguration we observe may involve $100 - 1000$ polarons, depending on the size of the domains. Taking an individual polaron mass of $m_p \simeq 3\ m_e$, we may have $m^* \sim 3 \times 10^3\ m_e$. For two domains to fuse, the electrons collectively move a distance of approximately elementary crystal one unit cell, $x \simeq 0.3\ nm$. At $4\ K$ this gives an attempt frequency of $\nu = \sqrt{\frac{k_B T}{2\pi^2 m^* x^2}} \sim 3 \times 10^{11}\ s^{-1}$. For comparison, the frequency of oscillation arising from a level spacing E_0 in a system of 1000 electrons trapped within a square well potential $\nu = \frac{E_0}{h} = h/8mL^2$. For a well width of $L \sim 0.3\ nm$, we obtain a similar value of $\nu \sim 10^{11}\ s^{-1}$.

With this values of ν, assuming a barrier width of $1\ nm$, and an experimentally determined $E_B = 2\ meV$, gives an estimated $R_q \simeq 3 \times 10^{-3}\ s^{-1}$. The rate estimate is very sensitive to the values in the exponent. However, the reasonable agreement between the experimentally measured $R_q = 2.2 \pm 0.3 \times 10^{-4}\ s^{-1}$ and the estimated R_q above shows that macroscopic quantum tunnelling between domain configurations is plausible on this timescale for our system with reasonable input parameters. In this estimate we ignore dislocations in the domain structure that topologically hinder relaxation. Such topological defects are an emergent

property, which arise from the microscopic interactions that MQT does not describe. We also ignore the effects of pinning of electrons due to crystal lattice imperfections that may reduce the rate of relaxation.

2.4 Quantum Kramers theory of metastable decay

A natural model for domain reconfiguration dynamics in 1T-TaS2 is a succession of single escapes from local minima in the energy landscape towards the global minimum (ground state), approximated locally by a cubic potential, assuming also some degree of coupling to an external Ohmic bath. Since the experimental $\Gamma(T)$ is averaged over many different escape events at temperature T, we may apply the canonical single particle escape from a cubic potential with Ohmic dissipation in order to estimate its average parameters. This relatively simple model predicts the classical high temperature Arrhenius law as well as the crossover to a temperature independent quantum decay at some crossover temperature T_0. The former arises from classical thermally activated hopping over the potential barrier E_B and the latter from tunneling events in the presence of dissipation, which exponentially suppresses the tunneling probability. From the experimental crossover temperature $T_0 = 20\,K$ and crossover temperature range $\Delta T = 10\,K$, we obtain according to Ref.[23] the dimensionless Ohmic damping parameter $\alpha = 0.3$ and the ratio of the activation barrier energy over the curvature (energy scale) of the local metastable minimum $\frac{E_B}{\hbar\omega_0} \simeq 0.4 \sim 1.4$, which is inconsistent with the underlying assumption of this model $E_B/\hbar\omega_0 \gg 1$. Therefore, quantum Kramers theory of metastable decay cannot explain the relaxation dynamics observed in 1T-TaS2.

2.5 Details on the incoherent macroscopic tunneling of our model

Here we make the argument for the mechanism of the decay process in 1T-TaS2 being incoherent macroscopic tunneling. We observe in experiment that the system starts off in some non-equilibrium domain state. Afterwards, we observe a reconfiguration event on a timescale of $\sim 10^3\,s$, which is separated by a factor of 10^{15} from the timescale obtained from the Arrhenius fit to the experimental rate $\hbar/E_B \sim 1\,ps$. It follows that the system is stuck in the initial state for a long time before decaying to the next configuration. Therefore, only the local energy levels in the energy spectrum are relevant to the decay, the next level lower in energy from the initial level being the most important. This is why a local two-level system approximation is appropriate for each observed reconfiguration event. The whole relaxation process can thereby be thought of as a succession of local decays in a two-level system, which is characterized by the tunneling rate $\Gamma_q \sim \Delta^2/W$ (discussed below). This is shown schematically in Fig. 2f. First, we assume a two level system with a bare gap Δ. If we couple such a system to a two-level oscillator bath, the 2 levels will split into 4 and the gap is reduced by $\sim W$. If the number of oscillators tends to ∞, the 2 levels become bands with width W. In our consideration of both the quantum annealer, as well as 1T-TaS2, we assume a large enough W for the two energy bands to overlap. Therefore, the transitions of the oscillators of the bath are the cause of the incoherent macroscopic tunneling processes we are observing.

Now we investigate the consequences of the conjecture that incoherent macroscopic tunneling is the mechanism responsible for domain reconfiguration dynamics in 1T-TaS_2, driven by the Hamiltonian $H = H_S + H_I + H_B$, where H_S describes the polaronic system, H_B represents a generic harmonic oscillator bath at temperature T, described by the spectral density $S(\omega)$ and H_I the system-bath interaction. If a two-level system is chosen as an approximation for a single reconfiguration event from a higher to a lower energy level in the full polaronic spectrum, where $H_S = -(\epsilon\sigma^z + \Delta\sigma^x)/2$, where σ^i are Pauli matrices, ϵ is the bias towards the system being in one of the two levels represented by the 2 values of σ^z and Δ the matrix element for pure tunneling between the two levels, analytical results are known from literature[24–28]. According to Amin et al[25] we assume $H_I = Q \otimes \sigma^z$, where Q is described by the bath spectral density $S(\omega) = \int_{-\infty}^{\infty} dt e^{i\omega t}\langle Q(t)Q(0)\rangle$, where $\langle \dots \rangle$ denotes averaging over the bath degrees of freedom, and write their main result $\Gamma_q \sim \Delta^2/W$, where $W^2 = \int \frac{d\omega}{2\pi} S(\omega)$ is the bandwidth of the bath noise. Γ_q is not the exact result of Amin et al; it only represents the scale of the quantum decay rate they obtained for level transitions. If the system is initialized in some non-equilibrium state, the bath will of course drive the system towards its thermal equilibrium at temperature T, but the timescale of these thermally induced transitions is still determined by Γ_q. Transitions occur between different system-bath product states $|S\rangle|B\rangle$ and are characterized by an incoherent tunneling rate $\Gamma_q \sim \Delta^2/W$. For example, in a single transition $|S\rangle$ might stay the same, while $|B\rangle$ undergoes some change, which leads to dephasing or decoherence of the whole system-bath state. This means that it is entirely possible for $|S\rangle$ states to retain coherence and for us to not observe any transitions, even though meanwhile, the bath is dephasing the whole state. On the other hand, $|S\rangle$ might undergo a transition simply due to a transition in $|B\rangle$. This occurs when the two bands corresponding to the two spin states are overlapping, which means that for some energy region, $|S\rangle|B\rangle$ states are separated by the energy gaps between $|B\rangle$ states and not Δ. The rate for such transitions is Γ_q. According to the aforementioned equivalence assumption that 1T-TaS_2 (M) and the quantum annealer (P) are both governed by the same H, and the fact that we are able to reproduce the saturation of $R(T)$ in M with P, meanwhile observing similar reconfiguration processes in P as in M, we argue that the perturbation theory developed for P is also applicable to M.

Now we apply the developed perturbation theory to our experiment and simulations, which takes the system's energy landscape into account through considering the entire energy spectrum system and focusing on the transitions between different energy levels (Fig. 2f). We assume that when the STM tip measures a polaronic configuration, it measures the occupation number $\langle\psi_M|n_i|\psi_M\rangle$ for every atomic site i and is insensitive to the bath degrees of freedom, which represent the noise of the system. The unperturbed Hamiltonian (when the STM tip is present) is therefore the potential energy between polarons, the coupling to the bath, and the bath $(B(s)H_z + H_I + H_B)$, because they only contain n_i and bath operators. The unperturbed energy spectrum contains all the possible polaronic configurations ranked by potential energy, which are broadened into bands by the bath. Of course, a single STM measurement forces the system into one specific polaronic configuration, where the bath degrees of freedom remain unspecified. This means that the whole polaron-bath system can lie anywhere on one of the polaronic energy spectrum bands, where the bath degrees of freedom change across the band, but the polaronic configuration is the same. The perturbation is the kinetic energy of polarons $(A(s)H_x)$, which is always present but only contributes significantly when the STM is removed

or turned off. Since the time-scale τ_M for relaxation is so large compared to t_M, the probability for transitioning to another configurational state is $\ll 1$, thus justifying the perturbation theory approach.

The gap $\Delta = g(H)E$ is characterized by the overall energy scale of the system E, where $g(H)$ is now the dimensionless gap, which typically depends exponentially on the Hamiltonian parameters[29,30]. The energy scale of P, $E_P = 1.6 \pm 0.8\, \mu eV$, governing the low temperature relaxation dynamics of system P is set by the magnetic fields operating on the SQUIDs serving as qubits on the QPU[31], while $E_M = 3\ \pm 0.4\, meV$ was measured in the STM experiment. We also know from previous literature[32,33] that the spectral density of noise in both M and P is well described as $1/\nu$ noise at low frequencies, which we assume is the dominant effect of noise in both cases. In order to make the connection from the fundamental time-scale to quantum incoherent relaxation, we rewrite $\Gamma_q = \frac{\Delta^2}{W} = g(H)^2 \frac{E}{W} E$ in terms of the energy scale, as well as the measured time-scale $\tau_M \approx 1000\, s \approx 10^{15}\, t_0^M$, where $t_0^M = \frac{\hbar}{E_M} \approx 2\, ps$. If $\Gamma = 1/\tau$, then $\Gamma_M = 10^{-15} E_M/\hbar\ \sim 10^{-15}\, E_M$. The 15 orders of magnitude difference is therefore explained by the quantity $g(H)^2 \frac{E_M}{W_M} = 10^{-15}$. In order to estimate the ratio $\frac{E_M}{W_M}$ in system M, we turn to the definition $W^2 = \int \frac{d\omega}{2\pi} S(\omega)$[27] and turn to measurements of low-frequency voltage fluctuations[32], which suggest that we can approximate the spectral density of the external bath of M with $1/\nu$ behavior as $S_M(\omega) = \frac{2.6 * 10^{-8} e^2 V^2}{\frac{\omega}{2\pi} + 0.34\, Hz}$. Integrating in the range of $3 - 100\, Hz$ returns a value of $W_M \approx 0.3\, meV$. Therefore, $\frac{E_M}{W_M} \approx 10$, which leads to $g(H) \sim 10^{-8}$, which in turn leads to a gap $\Delta_M \sim 10\, peV$ between different polaronic configurational states. If we take the value of W_P from literature[33] and calculate $\frac{E_P}{W_P} \approx 10$, we obtain a similar value as $\frac{E_M}{W_M}$. If we combine the same $1/\nu$ shape of the noise spectrum in both P and M with a similar quantitative value of energy scale to noise bandwidth ratio, we can conclude that the influence of noise is the same in P and M.

3 Supplementary Notes 3

In order to simulate quantum melting of domains we need to first be able to map our model on to an actual D-Wave machine. In this paper we use the newly released Advantage6.1 quantum computer with 5436 physical qubits. In this chapter we present all the necessary steps required for a successful deployment of our model.

3.1 Applying the model on D-Wave's machine

The Hamiltonian that is simulated by the D-Wave machine takes the form of the transverse field Ising model

$$H_{DW} = -\frac{A(t)}{2} \sum_i \sigma_i^x + \frac{B(t)}{2} \left(\sum_{i<j} J_{i,j} \sigma_i^z \sigma_j^z + \sum_i h_i \sigma_i^z \right), \tag{11}$$

where $\sigma_i^{x,z}$ are Pauli matrices operating on a qubit q_i, $J_{i,j}$ are couplings between qubits q_i and q_j and h_i are the longitudinal external fields at q_i. The maximum value that $A(t)$ and $B(t)$ take is about $10\ h\ GHz$[34]. The parameter t has a range of $[0, t_a]$ and plays the role of time, where t_a is the annealing time with a maximum available value of $2000\ \mu s$. The forms of $A(s)$ and $B(s)$ will be addressed in the next subsection.

In order to map our model onto $\widetilde{H}$, we need to apply the transformation from spin to qubit variables $\sigma_i^z = 2q_i - 1$. We can directly map the simplified version of our model H_{int} onto the second term of H by setting $h_i = \sum_{j=i}^{N} Q_{i,j}/2$ and $J_{i,j} = Q_{i,j}/4$. Since our model takes place on a two-dimensional lattice, it is therefore equivalent to an extended two-dimensional transverse field Ising model.

The next step in deploying the model is embedding it on the D-Wave machine's architecture. Here we need to assess the connectivity graph between our logical qubits ($q_i$in H_{int}) G and the actual connectivity graph between physical qubits on the machine G'. If G is not a subgraph of G' then we need to do a process called minor-embedding. We impose additional constraints between certain physical qubits by setting the couplings between them to the maximum possible ferromagnetic value ($J = -1$, for example) and link them in a chain. This setup forces all the physical qubits in a chain to assume the same value. Each chain then represents a single logical qubit. This process allows us to connect a logical qubits to more logical qubits than the connectivity of G' would allow us to if we just used one physical qubit to represent a single logical qubit. In practice due to quench dynamics as well as temperature and other noise effects, chains sometimes break. This means that when physical qubits in a chain are measured, they do not all have the same value. A D-Wave machine outputs information regarding broken chains in the form of the chain break fraction, which is simply the number of broken chains divided by the number of all chains.

In order to embed a triangular lattice with only nearest neighbor interactions onto the D-Wave Advantage processor, we first turn to the notation of physical qubits (PQs) used by Boothby et al[35]. By taking $M(= 16)$ as the parameter for the actual machine, we can parametrize the coordinates of a qubit on the physical quantum processing unit (QPU) as (u, w, z, k), where the index of the qubit available to the user is calculated by using $z + (M-1)(k + 12(w + Mu))$. Dattani et al[36] developed a more intuitive qubit coordinate representation defined by $(x_D, y_D, z_D, i_D, j_D, k_D)$ with the mapping $(u, w, z, k) = (i_D, (1-i_D)x_D + i_D(M-1-y_D), (1-i_D)\left(M-2-y_D+\delta_{z_D,0}\right) + i_D\left(x_D - \delta_{z_D,0}\right), k_D + 2j_D + 4z_D(1-2i_D) + 8i_D)$. We obtain a triangular lattice by assigning a logical qubit (LQ) to either 1 or 2 physical qubits according to

$$(-3y_D, 3x_D + 3y_D)_{LQ} = [(x_D, y_D, 0,0,0,0)]_{PQ} \tag{12}$$

$$(-3y_D + 1, 3x_D + 3y_D)_{LQ} = [(x_D, y_D, 0,1,1,1)]_{PQ}$$

$$(-3y_D, 3x_D + 3y_D + 1)_{LQ} = [(x_D, y_D, 0,1,0,0), (x_D, y_D, 0,0,1,1)]_{PQ}$$

$$(-3y_D - 1, 3x_D + 3y_D + 2)_{LQ} = [(x_D, y_D, 1,0,0,0)]_{PQ}$$

$$(-3y_D, 3x_D + 3y_D + 2)_{LQ} = [(x_D, y_D, 1,1,1,1)]_{PQ}$$

$$(-3y_D - 1, 3x_D + 3y_D + 3)_{LQ} = [(x_D, y_D, 1,1,0,0), (x_D, y_D, 1,0,1,1)]_{PQ}$$

$$(-3y_D - 2, 3x_D + 3y_D + 4)_{LQ} = [(x_D, y_D, 2,0,0,0)]_{PQ}$$

$$(-3y_D - 1, 3x_D + 3y_D + 4)_{LQ} = [(x_D, y_D, 2,1,1,1)]_{PQ}$$

$$(-3y_D - 2, 3x_D + 3y_D + 5)_{LQ} = [(x_D, y_D, 2,1,0,0), (x_D, y_D, 2,0,1,1)]_{PQ},$$

where $x_D \in [0, M-1]$ and $y_D \in [0, M-1]$. For the actual Python code, we employed for calculating the embedding, see our GitHub[37]. The maximum size of the triangular lattice we are able to currently embed on the actual QPU includes 2008 logical qubits, which consist of 2673 physical qubits. During our calculations on the actual QPU, there were 17 unavailable logical qubits, which reduced our lattice size from 2025 to 2008 sites. Supplementary Fig. 22 shows the triangular lattice used in our calculations.

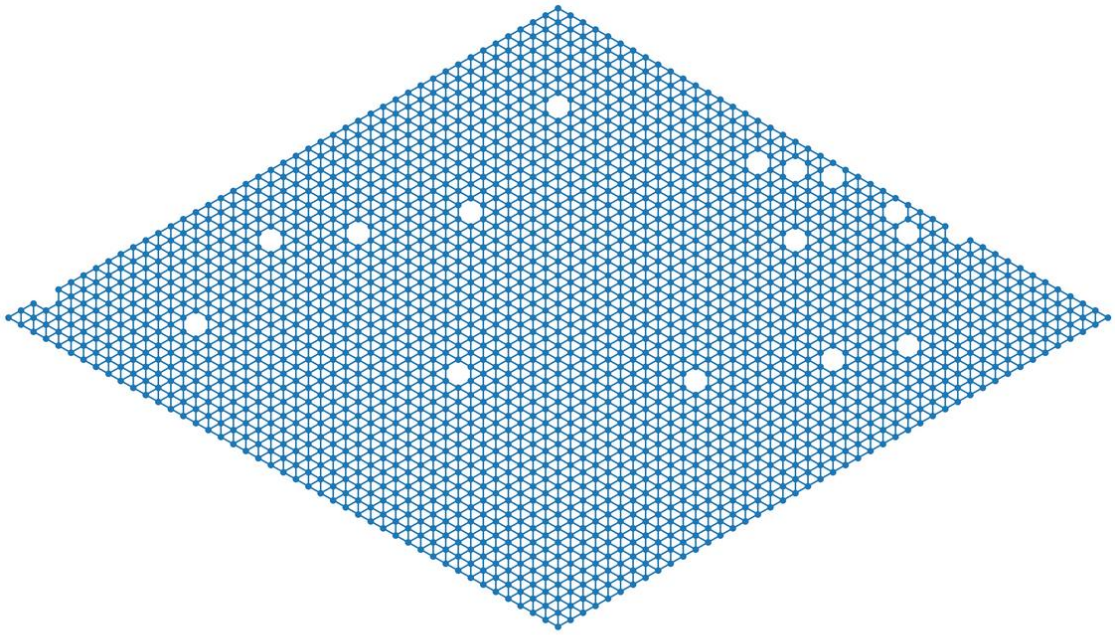

Supplementary Figure 22 | Triangular lattice consisting of 2008 sites with nearest neighbor interactions used in our calculations.

3.2 Introducing quantum dynamics by reverse annealing

In order to successfully model the experiment presented in this paper we needed to somehow introduce quantum dynamics into the system. We employed a feature provided by D-Wave called reverse annealing, where we can specify the annealing schedule. The time dependent functions $A(s(t))$ and $B(s(t))$ are both specified with a single function $s(t)$. We can then specify up to 12 points $[t, s(t)]$ in the range $[0, t_a] \times [0,1]$ from which D-Wave's Ocean™ software interpolates a polynomial for $s(t)$. We start off in the classical state ($s(0) = 1$), which is predetermined by us. We then reduce the value of $s(t)$ to s_{min} and bring it back up to 1 in t_a amount of time. In our simulations, we specified three points for the annealing schedule $(0,1), (t_a/2, s_{min}), (t_a, 1)$. The idea behind using this kind of schedule is to bring the system into a quantum superposition of different domain states close enough in energy so that a tunneling event between them becomes possible on the time scale used in our simulations. Supplementary Fig. 23 shows the predetermined annealing schedule used by the D-Wave Advantage6.1 used in our calculations. The temperature of the physical quantum processor was

fixed to $T_{DW} = 16 \pm 0.1\ mK$. Supplementary Fig. 24 shows the typical range of $s(t)$, $T_{eff} = k_B T_{DW}/A(s)$ and $A(s)/B(s)$ we used.

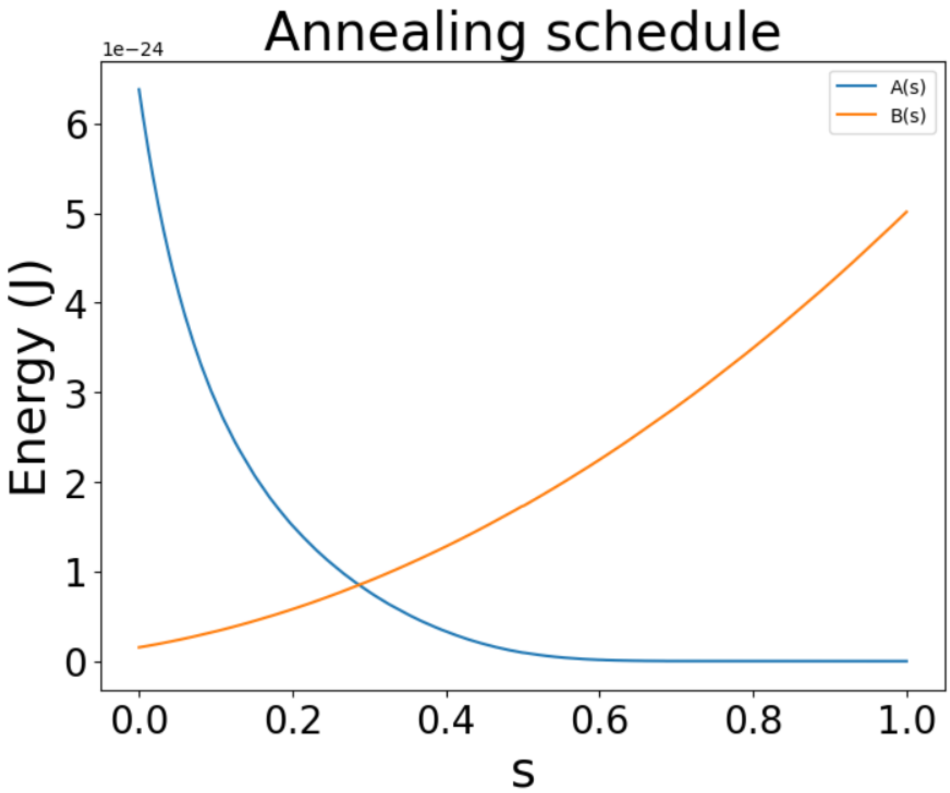

Supplementary Figure 23 | Predetermined annealing schedule used in our calculations.

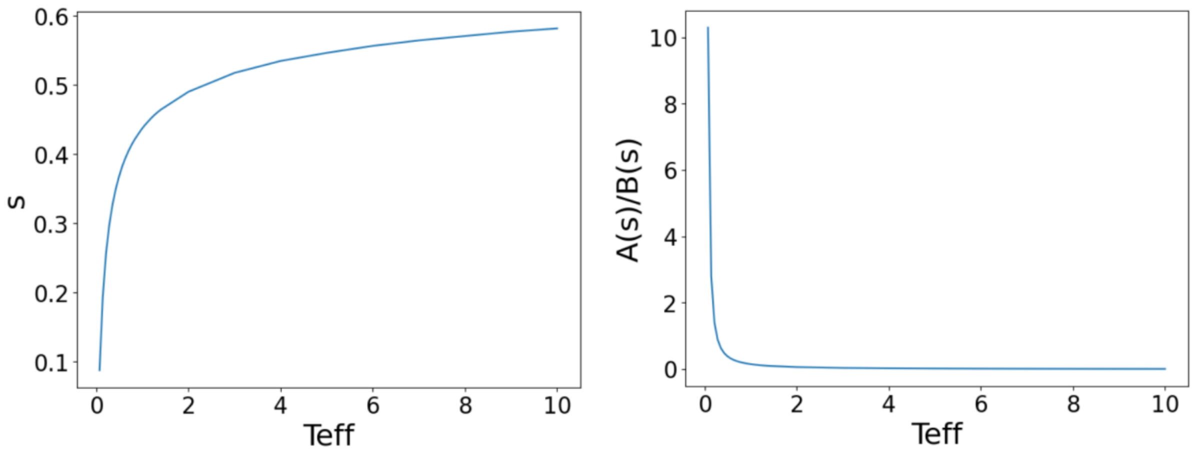

Supplementary Figure 24 | On the left, the dependence $T_{eff}(s)$ shown in the typical range used in our calculations. On the right, $\frac{1}{r} = A(s)/B(s)$ also shown in the typical range.

4 Supplementary Notes 4

4.1 Limitations by the annealer on the parameter space

After defining the parameters of our Hamiltonian under study $T_{eff} = k_B T_{DW}/A(s)$ and $r = JB(s)/A(s)$, we performed two experiments in order to ascertain the limitations on our parameter space. The first was designed in order to find the T_{eff} range in which we can operate. Supplementary Fig. 25 shows the dependence of the rate $R(T_{eff})$ as defined in the main text.

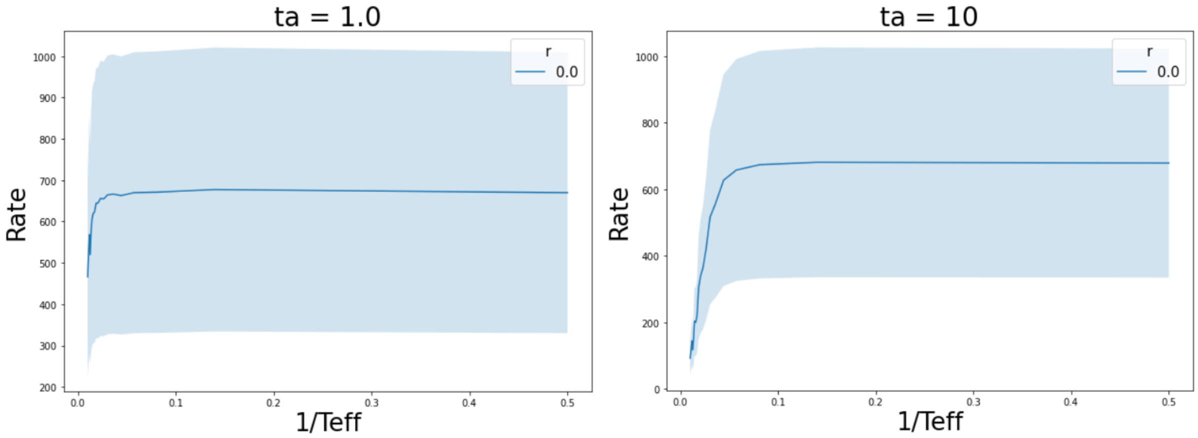

Supplementary Figure 25 | $R(T_{eff})$ dependence at $r = 0$, where a constant rate is expected shown for different annealing times $t_a = 1{,}10\ \mu s$. The shaded area shows the standard deviation when averaging over 1000 experiments.

We studied the value of $r = 0$, which is in the limit for which we know exactly what to expect. Since only the kinetic energy term is present with no interaction between polarons, we should effectively sample form random spatial configurations and the rate should therefore be temperature independent. This is exactly the case for $T_{eff} \in [2{,}10]$ as shown in Supplementary Fig. 25. For $T_{eff} > 10$ there is a sharp drop off of the rate, which is simply due to the fact of s_{min} becoming large enough for $A(s_{min})$ to be small enough not to be able to drive single qubit dynamics in the system within our measurement time scale. Simulations are therefore useless in this regime. On the other hand, the smallest T_{eff} at a certain value of r is set by the value of s and $J = 1$. In other words, at every $T_{eff}(s)$ we can tune r from $B(s)/A(s)$ to 0 by tuning J from 1 to 0, which is the allowed range on a D-Wave machine. The smallest possible non-zero r with $J = 1$ is $\frac{B(0)}{A(0)} \approx 0.0241$, which leads to a smallest $T_{eff}(0) \approx 0.0346$. When s increases from 0 to 1, $A(s \to 1) \to 0$, then $r \to \infty$ and $T_{eff} \to \infty$, but we have already found the upper limit of s above. We chose to investigate the range $\frac{B(s)}{A(s)} \in [0.1{,}90]$, which leads to a temperature range $[0.14{,}10]$ at $r = 0.1$ and $[10{,}10]$ at $r = 90$, thereby covering the whole parameter space currently available on the annealer.

4.2 Phase diagram calculation details

We calculated the phase diagram by sampling $\beta H_S = \frac{1}{T_{eff}}(-H_x + rH_z)$, where $r = [0.0, 0.1, 0.2, 0.3, 0.4, 0.5, 0.6, 0.7, 0.8, 0.9, 1.0, 1.1, 1.2, 1.3, 1.4, 1.5, 1.6, 1.7, 1.8, 1.9, 2.0, 3.0, 4.0, 5.0, 6.0, 7.0, 8.0, 9.0, 10.0, 20.0, 30.0, 40.0, 50.0, 60.0, 70.0, 80.0, 90.0, 100.0]$ and $T_{eff} = [0.07, 0.14, 0.21, 0.28, 0.35, 0.42, 0.49, 0.56, 0.63, 0.7, 0.77, 0.84, 0.91, 0.98, 1.05, 1.12, 1.19, 1.26, 1.33, 1.4, 2, 3, 4, 5, 6, 7, 8, 9, 10]$. The initial state was a fully occupied lattice, as shown in Supplementary Fig. 26.

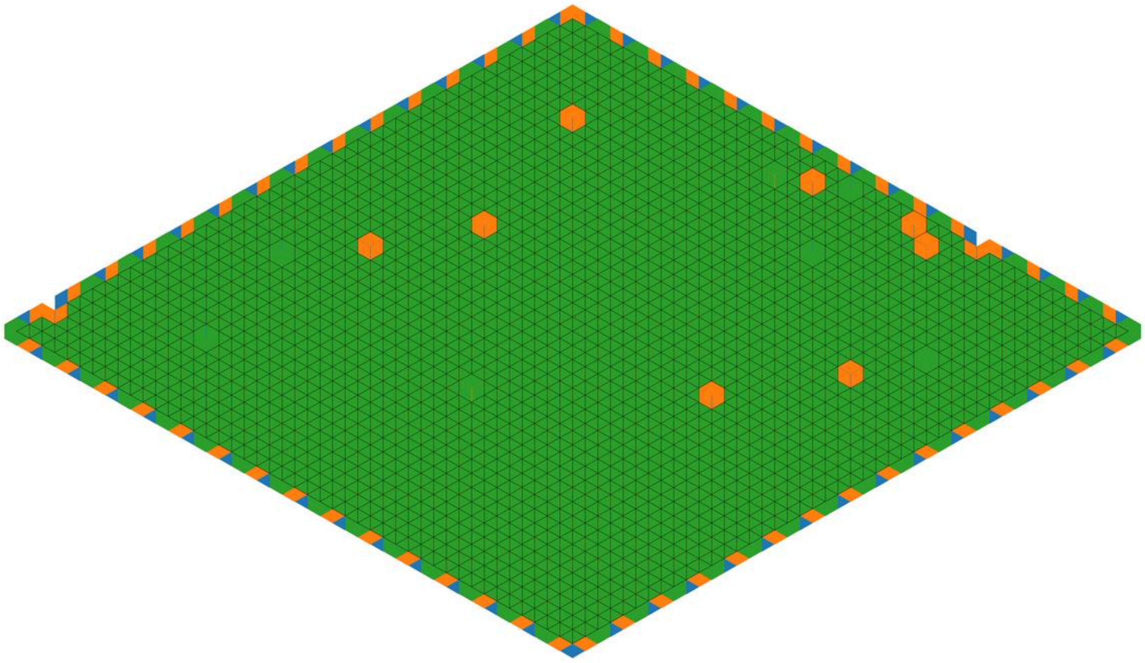

Supplementary Figure 26 | Fully occupied triangular lattice of polarons, used as the initial state for phase diagram calculations.

The interaction in QUBO language was set to $Q_{i,j} = 4$ for nearest neighbors and to $Q_{i.i} = -4$ for self-interaction. We then scaled $Q_{i,j}$ appropriately with J. We performed the reverse annealing protocol described previously 50 times, where the input state for the next iteration was the output of the previous. Supplementary Figs. 27 and 28 show the measured average rate of all the iterations for two annealing times $t_a = 1{,}10\ \mu s$.

In both cases we observe the phase transition from low-temperature ordering processes, which reduce the rate close to 0, to high temperature dynamics, where the system effectively samples from random distributions of polarons. This is why the rate saturates to a plateau, which is also the case at $r = 0$, where the system samples from random states at all temperatures. The main difference between $t_a = 1\ \mu s$ and $t_a = 10\ \mu s$ dynamics is in the low temperature ordering processes. In the former, the system typically does not order like in the latter case. While it is clear from configuration snapshots that at $t_a = 1\ \mu s$ the system samples the same kind of low energy states as in the $t_a = 10\ \mu s$ case, the latter case remains in the ground state or close by in energy. On the other hand, the former case is driven continuously from the ground state into

some excited state with domain walls. We conjecture that due to a fast enough annealing schedule, diabatic processes are responsible for transitions into higher energy states. This is not something we observed in 1T-TaS_2 experiments, which is why we decided not to focus on diabatic processes, but on the stable relaxation toward the ground state. Both plots also nicely show how the transition temperature increases when r increases.

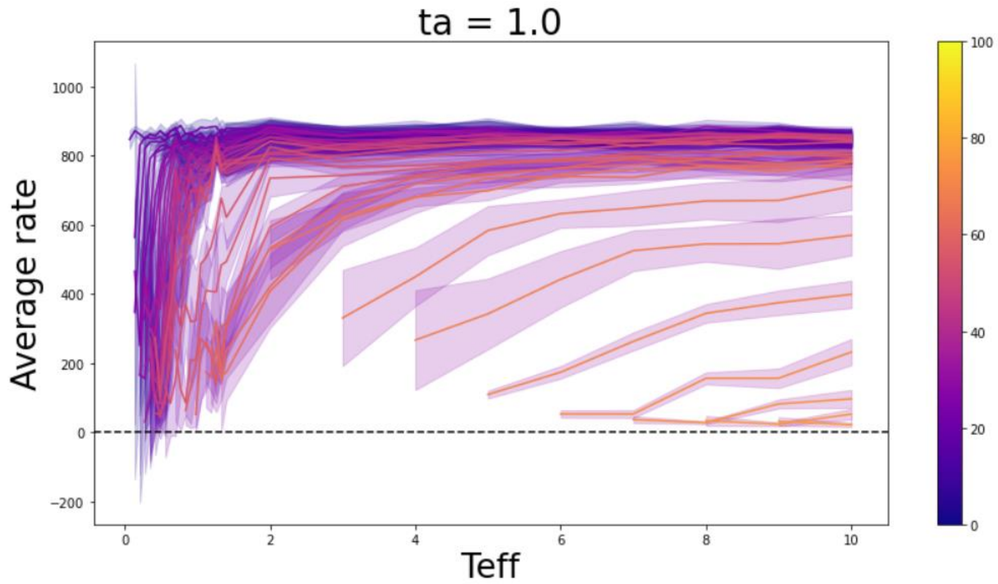

Supplementary Figure 27 | Average rate of polaronic movement versus T_{eff} for $t_a = 1\ \mu s$. The shaded area shows the standard deviation when averaging over 1000 experiments.

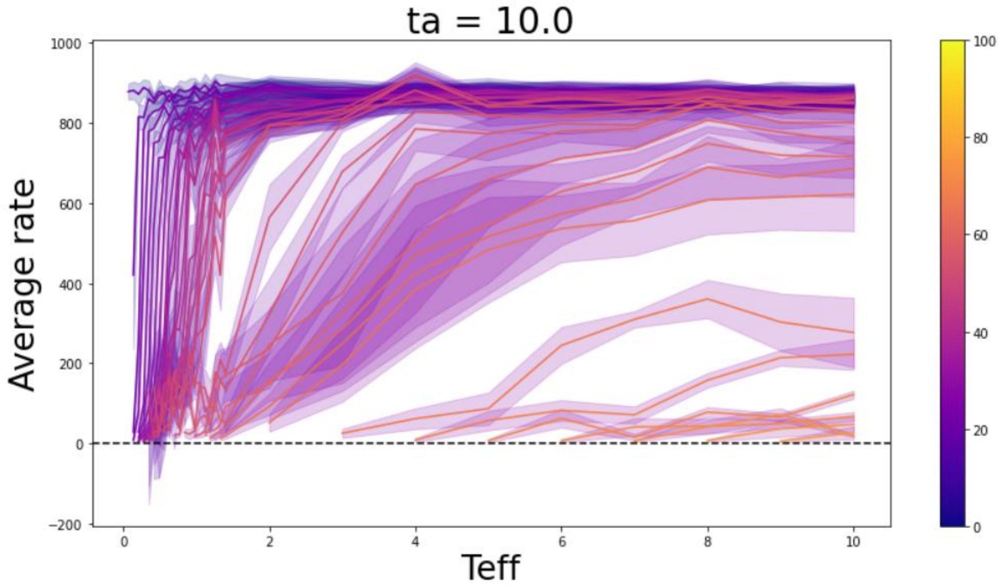

Supplementary Figure 28 | Average rate of polaronic movement versus T_{eff} for $t_a = 10\ \mu s$. The shaded area shows the standard deviation when averaging over 1000 experiments.

The transition temperature was measured by using the "blue order parameter", which is defined as the fraction of polarons in the positions of the blue colored $1/3$ triangular lattice as drawn in Fig. 2a of the main text. This turns out to be the ground state for the particular realization of our model on the D-Wave machine, separated by a very small energy difference from the orange and green ground states. For an ideal system, there is no difference between the 3 ground states. Therefore, if the blue order parameter grows to $1/3$, the system is in the ordered phase, and is significantly less in the gaseous phase where these blue lattice sites are occupied randomly.

Supplementary Fig. 29 shows the blue order parameter dependence on T_{eff}, with the vertical dashed lines representing our determined transition temperature for a specific r. The chosen transition temperatures were plotted versus r and a linear fit was done in order to extrapolate the transition temperature at higher values of r as shown in Supplementary Fig. 30. See main text for the discussion.

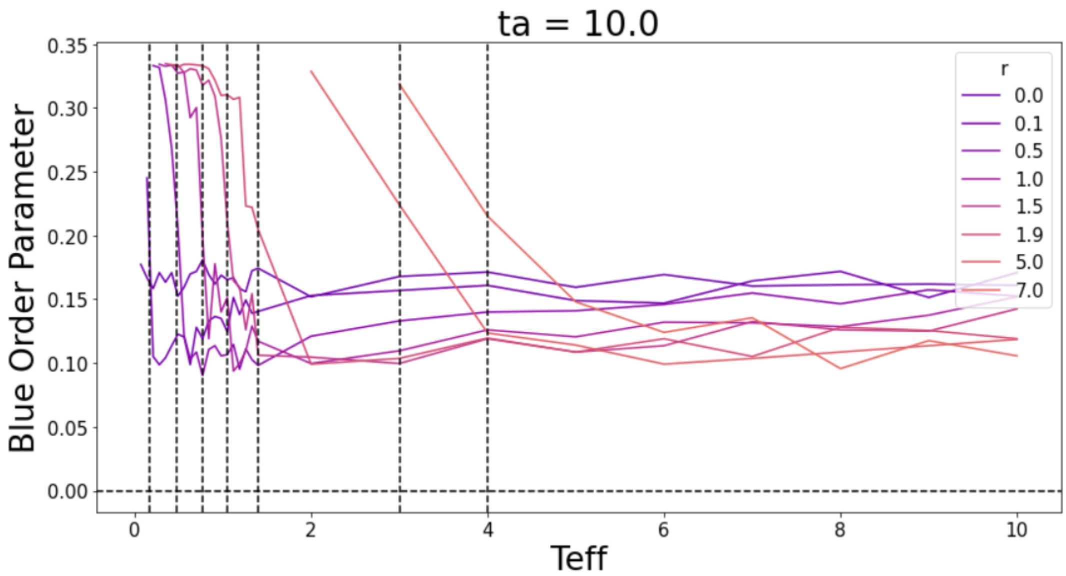

Supplementary Figure 29 | Blue order parameter versus T_{eff} for $t_a = 10\ \mu s$. The legend in the top right corner shows the values of r for which we measured the transition temperature from the low temperature ordered phase, to the high temperature gaseous phase. The vertical dashed lines show our choice of the transition temperatures by hand. The horizontal dashed black line is a guide to the eye for the value 0 of the blue order parameter.

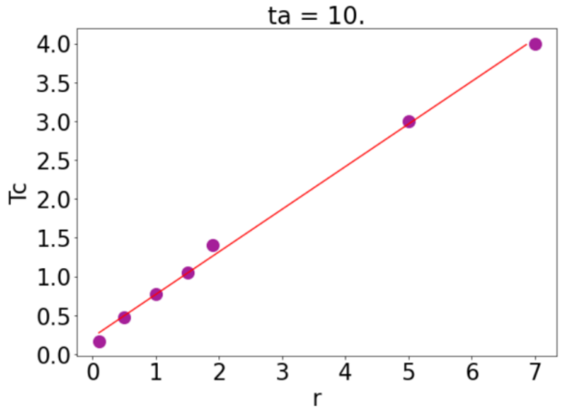

Supplementary Figure 30 | Transition temperature T_c dependence on r and a linear fit to the data $T_c = 0.55\, r + 0.22\ (1 \pm 5\%)$.

4.3 Relaxation measurements

In order to simulate the experiment on D-Wave's quantum computer, we first initialized a triangular lattice with 2008 sites in a domain state of a $1/3$ filled polaronic lattice shown in Fig. 2c (leftmost) and then performed a series of 50 reverse anneals. The initial domain state was obtained by a single reverse anneal from a fully occupied classical state at a high value of r. The output of the measured state after one reverse anneal was used as the input for the next. This procedure serves as an analogue to the actual real-world experiment in 1T-TaS_2. The initial polaronic configuration is also in a domain state of a $1/13$ filled polaronic lattice. We have

shown already in Ref.[16] that the behavior of polaronic lattices with different fillings is qualitatively the same. The initial domain state is then scanned with a scanning tunneling microscope (STM) sequentially in time and a relaxation to the $1/13$ ground state is observed after some time. The reverse annealing process on the D-Wave machine as performed in our case simulates what happens between two sequential scans. We therefore assume that the STM scan acts as a quantum measurement on a quantum state and collapses the wave function into a state in the classical number operator basis (n_i).

However, the true Hamiltonian of the system is quantum in the sense that a state in the number operator basis is not its eigenstate. In our simulations on D-Wave's machine the true quantum Hamiltonian is limited to the two-dimensional extended transverse field Ising model due to the physical architecture of the machine as described before. H_{int}, which represents charged classical polarons in our model of 1T-TaS$_2$, can be mapped from the number operator (n_i) basis to the spin basis (σ_i^z). The transverse field term (σ_i^x) therefore introduces non-trivial quantum effects such as tunneling between different configurations of qubits (polarons). However, we would like to point out that the transverse field in our case serves only as a means of introducing non-trivial quantum dynamics and we conjecture that the system would behave similarly with other forms of non-commuting terms added to H_{int}. Investigating this conjecture is out of the scope of this paper. Supplementary Fig. 31 shows typical rate dependence on time, which is just the index of the reverse annealing iterations multiplied by the t_a is which it was performed.

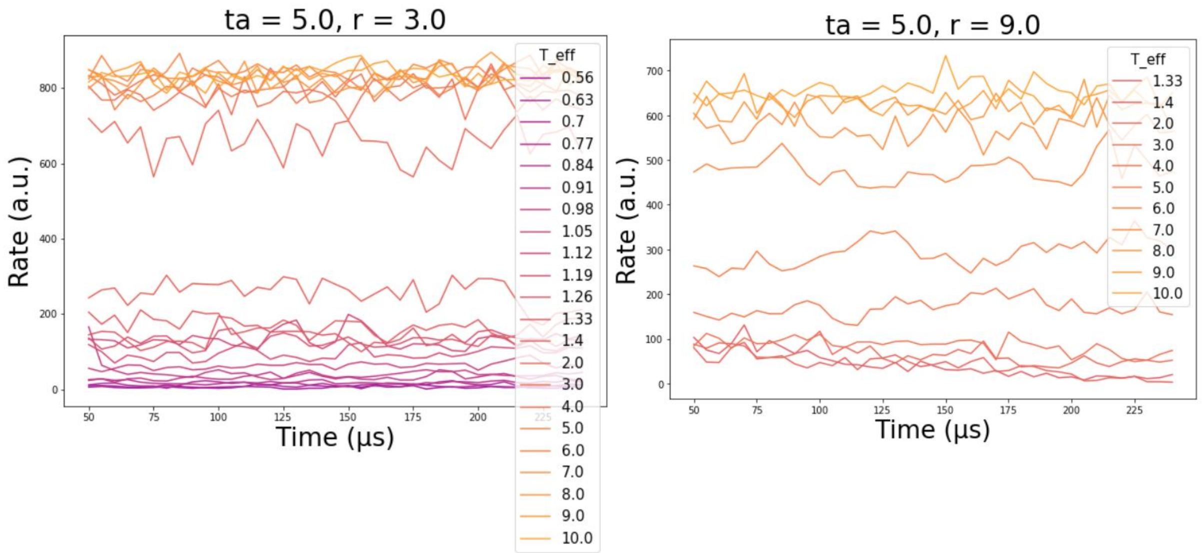

Supplementary Figure 31 | Rate as measured after every reverse annealing iteration vs time in the case of $r = 3$ (left) and $r = 9$ (right). Both datasets were obtained with $t_a = 5\ \mu s$.

There is a clear distinction between the low-temperature ordering dynamics and high-temperature random reconfigurations. It is also clear how the rate saturates to 0 at the lowest temperatures, because the system has reached the ground state as well as in the case of the highest temperatures, because the rate between two random configurations is on average the same. The $r = 9$ case shows a much slower rate relaxation as the $r = 3$ case at low T_{eff}, meaning that the system requires more reverse annealing iterations and hence more time in order to reach the ground state. This is due to the sufficiently large gap between states during the reverse annealing process, which suppresses the rate. This suppression of relaxation can

best be seen in Supplementary Fig. 32, which shows the energy measured at the end of each reverse anneal versus time as well as the exponential fits made to it and the extracted relaxation times dependence on r. The dependence is $\tau(r) = A * \exp\left(\frac{r}{r_0}\right) + B$, where $A = 2.6 \pm 1.2$, $B = 6.6 \pm 2.2$ and $r_0 = 2.1 \pm 0.3$.

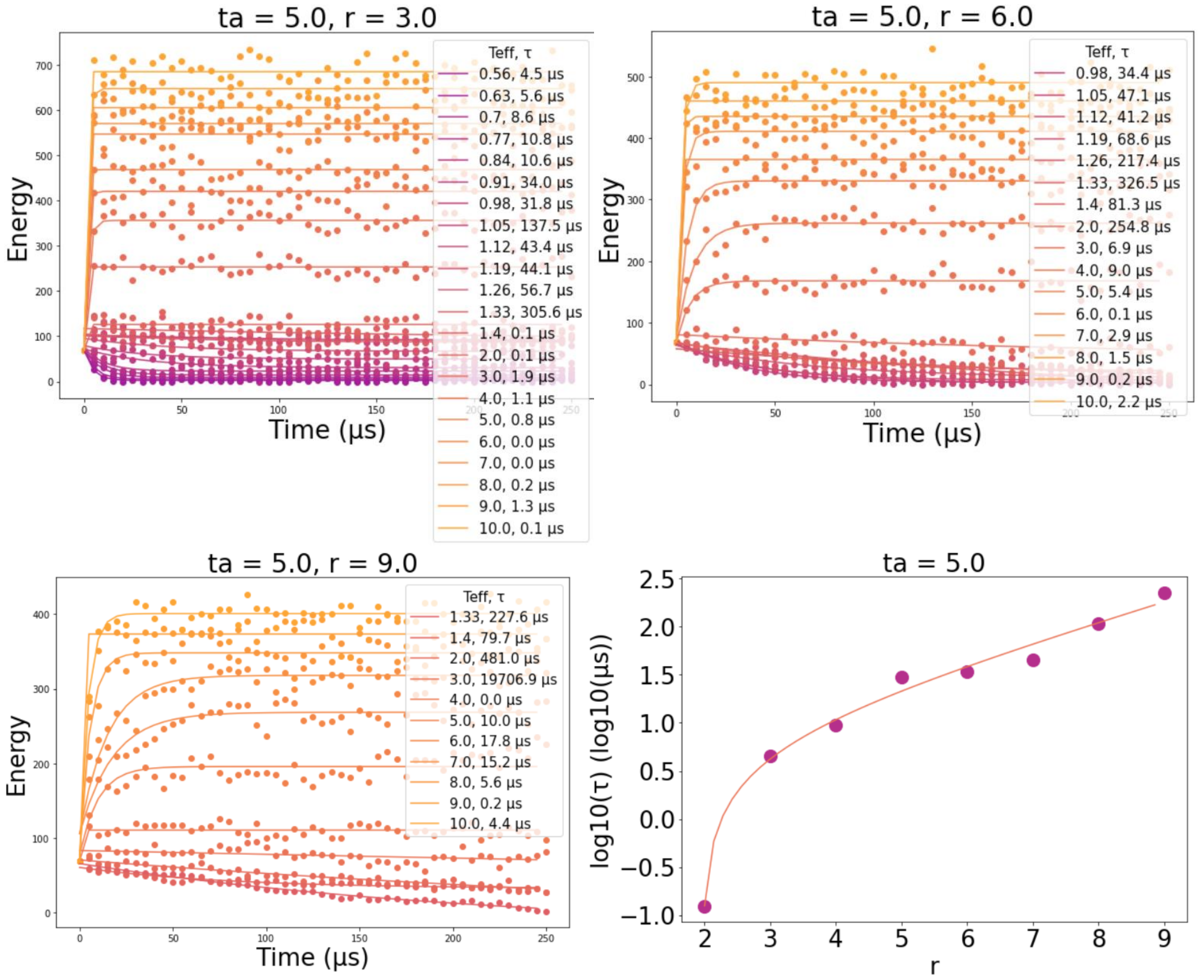

Supplementary Figure 32 | Energy measured after every reverse annealing iteration vs time in the case of $r = 3$, $r = 6$ and $r = 9$. All datasets were obtained with $t_a = 5\ \mu s$. The bottom right plot shows the $\tau(r)$ dependence, extracted from exponential fits in the other 3 plots in the figure, and its exponential fit (given in corresponding text).

The average rates at values of r other than the one presented in the main text are shown in Supplementary Fig. 33. At high T_{eff}, all rates saturate to the same value, determined by the average rate between two random configurations. At higher values of r, the rate saturates at low T_{eff} due to the system not yet reaching the ground state within the time window of our simulation. However, for lower values of r, the average rate tends more towards 0, which is inconsistent with our experimental observations. We emphasize here that if the time window of our simulation, or in the actual experiment would be long enough so that the system reaches the ground state, then the average rate would always be 0. The reason why it is not 0 in any case is simply due to the fact that the system has not yet reached the ground state. This is the reason we chose the value of $r \geq 9$ in the main text, as it most closely resembles the experimental plot of $R(T)$. The actual values of r and T_{eff} which correspond to sufficiently slow relaxation events have to be extrapolated from the fit in Supplementary Fig. 32. There is also an outlier feature

shown in Supplementary Fig. 33 at $r = 2$, which we would like to point out. These features do occur sometimes and are the result of the system switching from relaxing into the "orange" ground state to relaxing into the "blue". The system is predominantly ordered according to the orange ground state and then tunnels into the blue ground state, suggesting a non-zero tunneling matrix element between these two states. This has no bearing on our results, but is a possible avenue for future work.

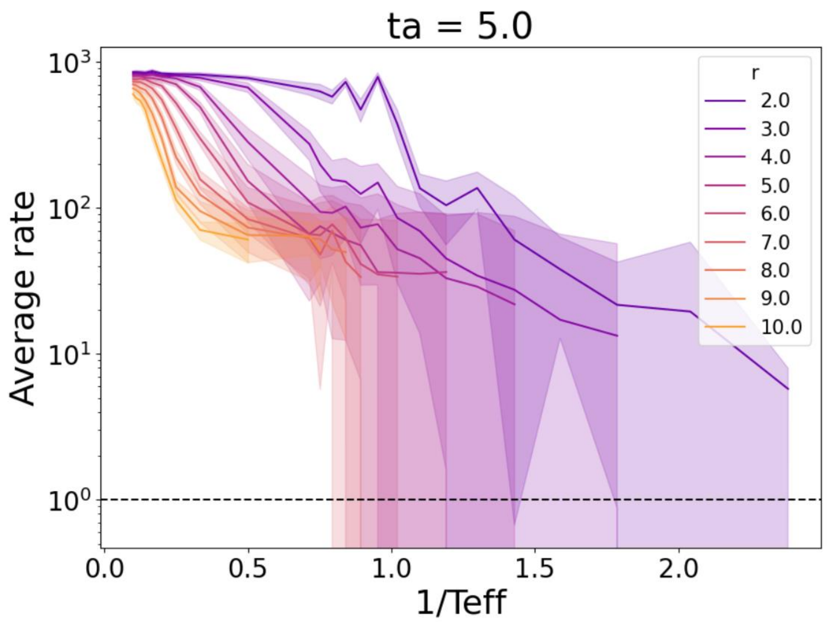

Supplementary Figure 33 | Average rate versus T_{eff} for different values of r listed in the top right legend. The shaded area shows the standard deviation when averaging over 1000 experiments.

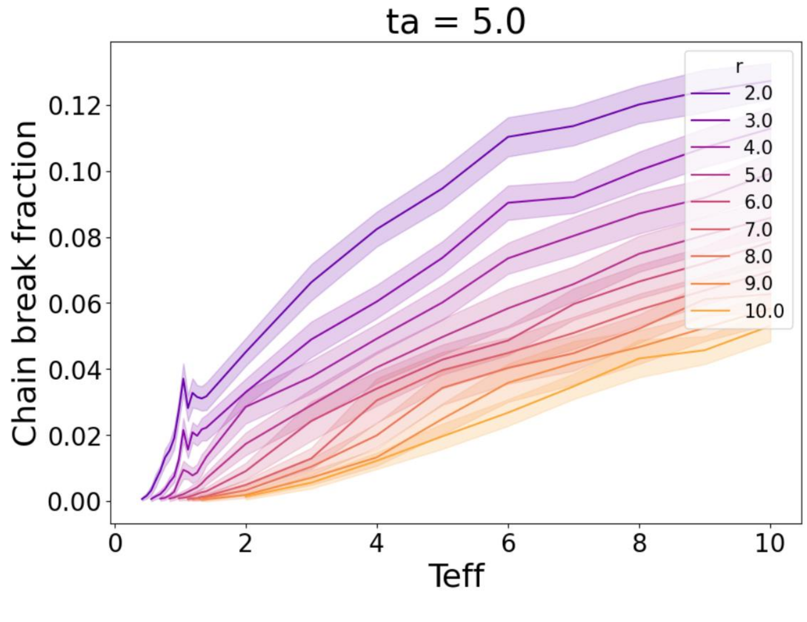

Supplementary Figure 34 | Average chain break fraction versus T_{eff} for different values of r listed in the top right legend. The shaded area shows the standard deviation when averaging over 1000 experiments.

There is an important quantity to study within the scope of our simulation, in order to check whether we are not introducing any artefacts into the simulation. The chain break fraction (CBF) is defined as the ratio of the number of broken chains and the number of logical qubits in the system. A broken chain is a logical qubit comprised of more than 1 physical qubit, where

the physical qubits are not all measured to be the same value. For more details, see section "Applying the model on the D-Wave's machine". In our embedding of the problem on the D-Wave machine, we use 665 out of 2008 logical qubits as chains of 2 physical qubits, which means that the maximum fraction of faulty qubits is 0.331. Supplementary Fig. 34 shows the average CBF for the cases shown in Supplementary Fig. 33. It does reach substantial values at higher T_{eff}, but it does not come close to the maximum value of 1. For low T_{eff} dynamics it is even heavily suppressed, which is why we are confident in the validity of the physics presented in this work.

Finally, we also determine the parameters for H, which reproduce this 15 orders of magnitude separation in time-scales by repeating the same consideration also for P. From P we obtain $\tau_P \approx 1 - 200\ \mu s$. In order to reach the same timescales as in M we estimate $r \sim 58$, which leads to a saturation of the rate at temperatures $T_{eff} \lesssim 32$ according to exponential fit extrapolation done in Supplementary Fig. 32 and Supplementary Fig. 30, respectively.

5 Supplementary References

1. Stojchevska, L. et al. Ultrafast switching to a stable hidden quantum state in an electronic crystal. Science 344, 177–180 (2014).
2. Ma, L. et al. A metallic mosaic phase and the origin of Mott-insulating state in 1T-TaS 2. Nat. Commun. 7, 1–8 (2016).
3. Cho, D. et al. Nanoscale manipulation of the Mott insulating state coupled to charge order in 1T-TaS2. Nat. Commun. 7, 1–6 (2016).
4. Vaskivskyi, I. et al. Fast electronic resistance switching involving hidden charge density wave states. Nat. Commun. 7, 1–6 (2016).
5. Vaskivskyi, I. et al. Controlling the metal-to-insulator relaxation of the metastable hidden quantum state in 1T-TaS 2. Sci. Adv. 1, e1500168 (2015).
6. Yu, Y. et al. Gate-tunable phase transitions in thin flakes of 1T-TaS2. Nat. Nanotechnol. 10, 270–276 (2015).
7. Cho, D. et al. Correlated electronic states at domain walls of a Mott-charge-density-wave insulator 1T-TaS2. Nat. Commun. 8, 1–6 (2017).
8. Cho, D., Cho, Y. H., Cheong, S. W., Kim, K. S. & Yeom, H. W. Interplay of electron-electron and electron-phonon interactions in the low-temperature phase of 1T-TaS2. Phys. Rev. B - Condens. Matter Mater. Phys. 92, 1–5 (2015).

9. Gerasimenko, Y. A., Karpov, P., Vaskivskyi, I., Brazovskii, S. & Mihailovic, D. Intertwined chiral charge orders and topological stabilization of the light-induced state of a prototypical transition metal dichalcogenide. Npj Quantum Mater. 4, 32 (2019).
10. Suzuki, A., Koizumi, M. & Doyama, M. Thermal evidences for successive CDW phase transitions in 1T-TaS2. Solid State Commun. 53, 201–203 (1985).
11. Núñez-Regueiro, M. D., Lopez-Castillo, J. M. & Ayache, C. Thermal conductivity of 1T-TaS2 and 2H-TaSe2. Phys. Rev. Lett. 55, 1931–1934 (1985).
12. Ravnik, J. et al. A time-domain phase diagram of metastable states in a charge ordered quantum material. Nat. Commun. 12, 2323 (2021).
13. Elliott, S. R. A.c. conduction in amorphous chalcogenide and pnictide semiconductors. Adv. Phys. 36, 135–217 (1987).
14. Karpov, P. & Brazovskii, S. Modeling of networks and globules of charged domain walls observed in pump and pulse induced states. Sci. Rep. 8, 1–7 (2018).
15. Mihailovic, D. et al. Ultrafast non-thermal and thermal switching in charge configuration memory devices based on 1T-TaS2. Appl. Phys. Lett. 119, (2021).
16. Vodeb, J. et al. Configurational electronic states in layered transition metal dichalcogenides. New J. Phys. 21, (2019).
17. Alexandrov, A. S. & Mott, N. F. Polarons and Bipolarons. (WORLD SCIENTIFIC, 1996). doi:10.1142/2784.
18. Vodeb, J. et al. Theoretical Modeling of the Non-equilibrium Amorphous State in 1T-TaS2. J. Supercond. Nov. Magn. (2019) doi:10.1007/s10948-019-5028-1.
19. Balducci, F., Gambassi, A., Lerose, A., Scardicchio, A. & Vanoni, C. Localization and Melting of Interfaces in the Two-Dimensional Quantum Ising Model. Phys. Rev. Lett. 129, 120601 (2022).
20. Daley, A. J. et al. Practical quantum advantage in quantum simulation. Nature 607, 667–676 (2022).
21. Vodeb, J. et al. Configurational electronic states in layered transition metal dichalcogenides. New J. Phys. 21, 083001 (2019).

22. Gerasimenko, Y. A. et al. Quantum jamming transition to a correlated electron glass in 1T-TaS2. Nat. Mater. 18, 1078–1083 (2019).
23. Grabert, H., Olschowski, P. & Weiss, U. Quantum decay rates for dissipative systems at finite temperatures. Phys. Rev. B 36, 1931–1951 (1987).
24. Leggett, A. J. et al. Dynamics of the dissipative two-state system. Rev. Mod. Phys. 59, 1–85 (1987).
25. Amin, M. H. S. & Averin, D. V. Macroscopic Resonant Tunneling in the Presence of Low Frequency Noise. Phys. Rev. Lett. 100, 197001 (2008).
26. Amin, M. H. S., Love, P. J. & Truncik, C. J. S. Thermally Assisted Adiabatic Quantum Computation. Phys. Rev. Lett. 100, 060503 (2008).
27. Amin, M. H. S., Averin, D. V. & Nesteroff, J. A. Decoherence in adiabatic quantum computation. Phys. Rev. A 79, 022107 (2009).
28. Amin, M. H. S., Truncik, C. J. S. & Averin, D. V. Role of single-qubit decoherence time in adiabatic quantum computation. Phys. Rev. A 80, 022303 (2009).
29. Knysh, S. Zero-temperature quantum annealing bottlenecks in the spin-glass phase. Nat. Commun. 7, 12370 (2016).
30. Isakov, S. V. et al. Understanding Quantum Tunneling through Quantum Monte Carlo Simulations. Phys. Rev. Lett. 117, 180402 (2016).
31. QPU-Specific Characteristics — D-Wave System Documentation documentation. https://docs.dwavesys.com/docs/latest/doc_physical_properties.html#doc-qpu-characteristics.
32. Salgado, R. et al. Low-frequency noise spectroscopy of charge-density-wave phase transitions in vertical quasi-2D 1T-TaS_2 devices. Appl. Phys. Express 12, 037001 (2019).
33. Whiticar, A. M. et al. Probing flux and charge noise with macroscopic resonant tunneling. Phys. Rev. B 107, 075412 (2023).
34. D-Wave Systems Inc. Technical Description of the D-Wave Quantum Processing Unit. Tech. Descr. -Wave Quantum Process. Unit (2021).
35. Boothby, K., Bunyk, P., Raymond, J. & Roy, A. Next-Generation Topology of D-Wave Quantum Processors. Preprint at http://arxiv.org/abs/2003.00133 (2020).

36. Dattani, N., Szalay, S. & Chancellor, N. Pegasus: The second connectivity graph for large-scale quantum annealing hardware. Preprint at http://arxiv.org/abs/1901.07636 (2019).

37. Vodeb, J., Non-equilibrium quantum domain reconfiguration dynamics in a two-dimensional electronic crystal versus a quantum annealer, Embedding a triangular lattice on D-Wave, 10.5281/zenodo.11061074, 2024
